# Using Collaborative Model Building to Better Understand the Mechanisms of Alcohol-Involved Sexual Violence on College Campuses: Post Hoc Protocol

**DOI:** 10.2196/92071

**Published:** 2026-06-08

**Authors:** Michelle Dougherty, Jessica G Burke, Joseph Alejandro, Travis R Moore, Robert W S Coulter, Regina Futcher, Elizabeth Miller, Christina Mair

**Affiliations:** 1 Department of Behavioral and Community Health Sciences, School of Public Health, University of Pittsburgh, 130 DeSoto St, Pittsburgh, PA, 15261, United States, 1 412-624-3613; 2 ChildObesity180, Friedman School of Nutrition Science and Policy, Tufts University, Boston, MA, United States; 3 Department of Pediatrics, School of Medicine, University of Pittsburgh, Pittsburgh, PA, United States

**Keywords:** systems science, group model building, alcohol, sexual violence, college

## Abstract

**Background:**

The Collaborative Model Building Project to Understand Sexual Violence (CAMPUS) study seeks to address alcohol-involved sexual violence (AISV) by collaboratively developing an agent-based model (ABM) that can support the decisions of college campuses seeking to address this issue among students. As a first step toward ABM development, we used collaborative model building (CMB), an adaptation of group model building, to co-develop a causal loop diagram (CLD) depicting key causes and effects of AISV and opportunities for intervention. Our goal of cocreating a CLD that can be translated into an ABM to support intervention decision-making differentiates our approach from other participatory systems science studies.

**Objective:**

This paper provides a detailed, transferable post hoc protocol for using CMB to cocreate a CLD of AISV on college campuses that can be translated into an ABM to support intervention decision-making.

**Methods:**

Our approach consisted of 4 iterative phases that involved ongoing weekly discussion by the project’s core modeling team (CMT), consisting of researchers with systems science and subject matter expertise. In the first phase, we conducted 4 CMB sessions with 3 groups of college campus collaborators to develop 1 preliminary CLD each. Second, our CMT reviewed each variable and causal connection across the CLDs in consultation with peer-reviewed literature to help ensure eventual ABM translation. Third, the CMT combined 3 CLDs into one, identifying specific loops for review by collaborators. Fourth, we conducted a feedback session with collaborators and created a finalized CLD linked with intervention opportunities.

**Results:**

Between January 2023 and March 2025, we engaged with 39 collaborators in Allegheny County, Pennsylvania, across three groups: (1) college campus practitioners (eg, student life staff; n=8), (2) undergraduate students (n=12), and (3) a mix of practitioners and students (n=19). The final output of our process was a complete CLD with 26 loops containing 28 variables showing key AISV mechanisms and intervention opportunities. Analysis of results from the CAMPUS study, funded from September 2022 to June 2027, is ongoing, and results from aim 1 of the study are expected to be published in late 2026.

**Conclusions:**

Given our goal of using the finalized CLD for ABM development, our approach emphasized the selection of potentially measurable and modifiable variables and causal connections. Compared with other participatory systems science approaches, our approach also emphasized intervention identification and prioritization.

## Introduction

Complex systems science approaches enable the examination of how factors spanning different social-ecological levels interact with one another over time to create feedback loops and emergent properties (ie, dynamics that are a product of a system as a whole as opposed to any one individual factor) [1,2]. Of these methods, group model building (GMB) is particularly amenable to involving participants as study collaborators. Through this method, researchers and participants cocreate causal loop diagrams (CLDs) depicting how key factors related to a problem of interest causally relate to one another [3]. GMB has been used to understand how to intervene upon a range of complex health issues [4-11]. Thoroughly documenting GMB methods is important, as GMB is a long, multiphase process and there is substantial flexibility in how it can be implemented [12].

The Collaborative Model Building Project to Understand Sexual Violence (CAMPUS) study seeks to collaboratively develop an agent-based model (ABM) of alcohol-involved sexual violence (AISV) that can serve as a decision support tool to help college campuses address this issue among students. We used collaborative model building (CMB), which is similar to GMB but distinct in that it de-emphasizes training collaborators in systems thinking and focuses more heavily on eliciting input on key modifiable mechanisms, identifying opportunities for intervention, and refining CLD elements for translation into an ABM. The objective of this paper is to provide a post hoc protocol for using CMB to generate a CLD of AISV on college campuses that can be translated into an ABM.

## Methods

### CMB Overview

As shown in Table 1, the CMB process for the CAMPUS study took place over the course of 4 phases, beginning in January 2023 with the engagement of 3 groups of college campus collaborators and ending in March 2025 with the completion of a CLD integrated from all 3 groups. The scope of the CLD was limited to factors that college campuses could intervene upon to address AISV among their students. This scope guided many of the decisions that we made in developing and refining the CLD.

**Table 1.** Overview of collaborative model building phases for the Collaborative Model Building Project to Understand Sexual Violence (CAMPUS) study.

Phase	Period	Goal	Key steps	Output
Implementing CMB^a^ to produce preliminary CLDs^b^	January 2023 to February 2024	To engage 3 groups of college campus collaborators to illustrate the causes and effects of alcohol-involved sexual violence on college campuses	Planning CMB sessions Engaging collaborators Conducting CMB sessions	Three preliminary CLDs; 1 from each group
Refinement of individual CLDs by CMT^c^	February 2024 to September 2024	To review each variable and connection in consultation with published literature and to identify closed loops and corresponding narratives	Reviewing variables and connections Identifying loops	A “refined,” easier-todigest CLD for each group
CLD integration	September 2024 to Novemeber 2024	To combine 3 CLDs (1 from each group) into a unified CLD	Identifying common variables and loops	One integrated CLD
Finalization of CLD with collaborator feedback	November 2024 to March 2025	To elicit collaborator feedback on integrated CLD	Conducting collaborator feedback session Integrating feedback and quality-checking the final CLD Linking interventions to integrated CLD	A finalized CLD linked with example interventions

a CMB: collaborative model building.

b CLD: causal loop diagram.

c CMT: core modeling team.

In phase 1, we developed preliminary CLDs with each of the 3 groups of collaborators. Although phase 1 allowed for some refinement of the CLDs, there was not sufficient time to review all variables and connections in depth and pare each CLD down to depict only closed loops. Therefore, the goal of phase 2 was for the core modeling team (CMT) to review each variable and connection in consultation with published literature, identify closed loops and corresponding narratives, and produce a “refined,” easier-to-digest CLD for each group. In phase 3, we combined the CLDs from all 3 groups into 1 combined CLD, as the project ultimately aimed to create an ABM that incorporated the perspectives of all 3 groups of collaborators. In refining and integrating CLDs, we identified specific loops that we wanted our student and college practitioner collaborators to review. These loops generally included dynamics that we could not validate on the basis of existing peer-reviewed literature or our theoretical knowledge but nonetheless seemed important in light of the feedback we received during the CMB sessions in phase 1. Therefore, the goals of phase 4 were to seek collaborator feedback on specific loops in the integrated CLD, use this feedback to finalize the CLD, and link example interventions to this finalized version.

All phases of the CMB process were overseen by a CMT whose purpose was to employ systems science and subject matter expertise to produce a finalized CLD based on input from campus collaborators. The CMT consisted of a group of 7 faculty, postdoctoral fellows, and graduate students with expertise in alcohol, sexual violence, GMB, systems science, community-engaged research, human-centered design, and college health intervention implementation. Some members of the CMT identified as cisgender heterosexual men and women, and some identified as cisgender gay and bisexual men. Most identified as White, and a few identified as Asian. Some members of the CMT had lived experiences of sexual violence personally or through close friends and family. One member of the CMT had implemented several GMB projects before, and 4 members of the team had conducted GMB as part of a separate pilot project [13].

Through all phases of the CMB process, the CMT met once a week for 1 hour via Zoom (version 7.0.2; Zoom Communications Inc). During these weekly meetings, the CMT reviewed the materials and output from the CMB sessions and made decisions about session facilitation and model refinement through group consensus. The CMT sought to balance their knowledge as researchers with the input from college campus collaborators with lived experiences as students and practitioners interested in or working on sexual violence prevention. Details and examples of CMT activities, including how the CMT balanced their perspectives with that of collaborators, are presented in the sections below.

### Ethical Considerations

The CMB activities were determined not to constitute human subjects research by the Institutional Review Board (IRB) at the University of Pittsburgh (STUDY22080147). The minimal risk activities focused on the development of systems models through the aggregation of collaborators’ knowledge and perspectives rather than the collection of individual-level data for research purposes. The IRB’s assessment ensured compliance with ethical standards and guidelines, affirming that the CMB process involved collaborative, participatory methods that did not directly involve human subjects research protocols. Given this determination, informed consent was not necessary. We kept all input from collaborators confidential by not including identifying information in our reporting of results. Collaborators received US $75 per session, a US $25 bonus for attending 3 sessions, and a US $50 bonus for attending 4 sessions (up to US $375 total), in addition to US $15 per session to cover parking and transportation costs. All payments were made on university gift cards (similar to debit cards) after each session.

### Phase 1: Implementing CMB to Produce Preliminary CLDs

#### Planning CMB Sessions

In January and February 2023, the CMT prepared CMB session materials, beginning with solidifying the overarching goal of these sessions: to understand the key modifiable causal mechanisms associated with AISV on college campuses based on the perspectives of college campus collaborators. We selected session activities from Scriptapedia, an online compendium of GMB activities [14] and supplemented them with human-centered design activities from the Luma Institute [15]. We then developed preliminary session agendas to decide how best to sequence and facilitate these activities. We chose to conduct CMB over the course of multiple sessions to allow for CMT review and refinement between sessions and to avoid collaborator burnout or fatigue. We ultimately decided that we would have 4 sessions of 2 hours each, held in person, with 2 to 3 weeks between sessions, depending on the amount of work we anticipated for the CMT during these interim periods. On the basis of preliminary agendas, we ordered supplies and drafted materials (eg, Microsoft PowerPoint presentations and handouts), described further below. Some session materials were contingent on the output of prior sessions; thus, part of the work of the CMT between sessions consisted of preparing these materials.

#### Engaging CMB Collaborators

We engaged three groups of collaborators affiliated with undergraduate colleges in Allegheny County, Pennsylvania: (1) practitioners (eg, campus health educators, counseling, and student life staff), (2) undergraduate students, and (3) a mix of undergraduate students and practitioners. To recruit practitioners for group 1, we distributed a study flyer via contacts at undergraduate institutions throughout Allegheny County, Pennsylvania, using networks established through ongoing sexual violence prevention efforts [16]. We used this same network to recruit students for groups 2 and 3. Practitioners from the first group also assisted with recruitment for groups 2 and 3 by disseminating the study flyer through their networks. The flyer included a link to an eligibility survey on Qualtrics that assessed the demographics, institutional affiliations, and availability for all collaborators; professional roles for practitioners; and year of study, major, living situation (on campus or off campus), and extracurricular activities for students.

Compared to other groups, a smaller group of practitioners filled out the eligibility screening survey for group 1 (n=10). We invited all 10 to participate, and 8 joined at least 1 session. In contrast, 101 undergraduate students filled out the eligibility screening survey for group 2. Of these, we invited 21 students, seeking maximum diversity across institutions, demographic characteristics (age, race/ethnicity, gender identity, and sexual orientation), and other characteristics (eg, year of study). Students enrolled only in online courses were excluded. Of the 21 students that we invited, 12 participated in at least 1 session. For group 3, an additional 14 practitioners and 74 students filled out the eligibility screening survey and we invited 24 individuals to participate, once again seeking maximum heterogeneity in terms of demographics, institutional affiliations, and—for practitioners—professional roles. Of these 24 individuals, 9 practitioners and 10 students participated in at least 1 CMB session. For all 3 groups, recruitment took place approximately 2 weeks before the start of CMB sessions.

#### Conducting CMB Sessions

We conducted CMB sessions with group 1 (practitioners) in April to June 2023, with group 2 (students) in September to November 2023, and with group 3 (students and practitioners) in November 2023 to February 2024. Three members of our CMT facilitated all sessions: 2 faculty members and a doctoral student with expertise in alcohol, sexual violence prevention, systems science, human-centered design, qualitative research, and management of group dynamics. For most sessions and activities, the 2 faculty members took the lead on speaking and facilitation roles (eg, introducing activities and guiding conversations), while the doctoral student took notes, recorded sessions, and handled logistics (eg, taking attendance, distributing materials, and ensuring collaborators received incentive payments). One member of the facilitation team took photos throughout the sessions to document the session outputs. All sessions were conducted in a classroom or conference room with a projector screen in an academic building. Prior to each session, we sent a reminder to collaborators, along with directions (eg, where to park and how to get to the room where the session was taking place). The facilitation team met to do a dry run before each session.

**Figure 1.**
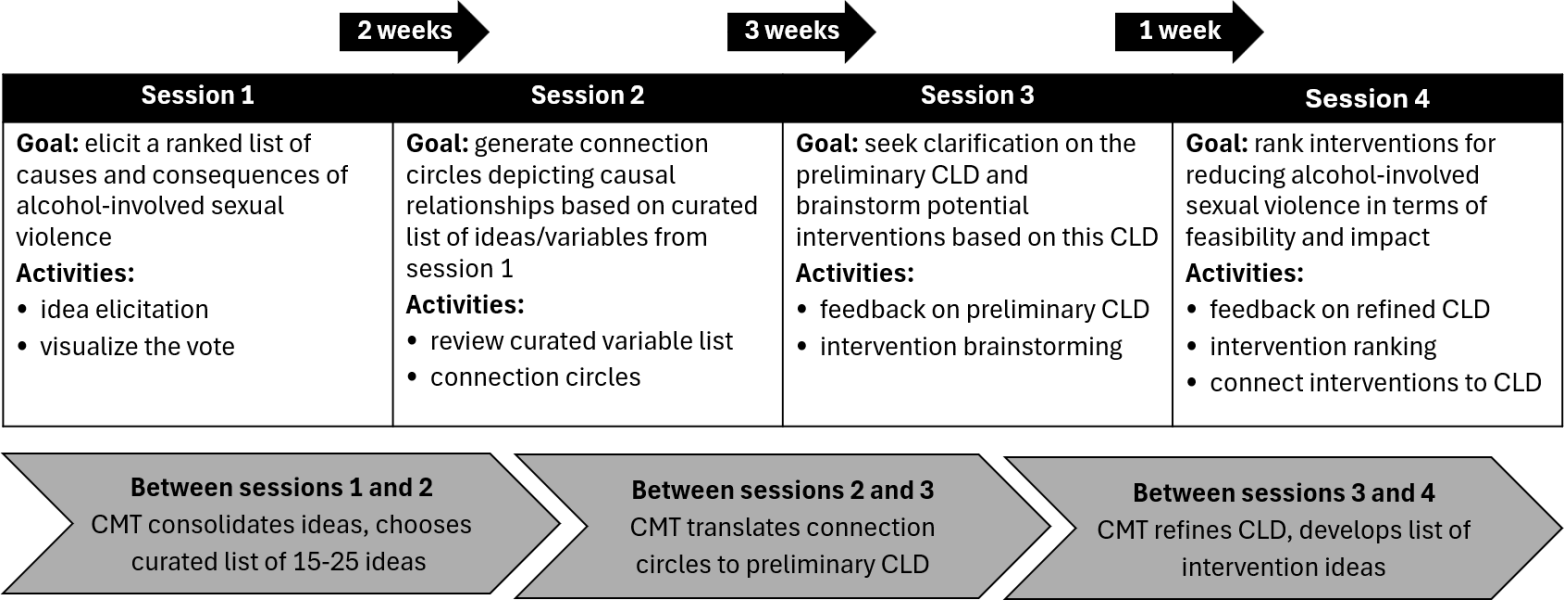
Overview of collaborative model building sessions. CLD: causal loop diagram; CMT: core modeling team.

Figure 1 provides an overview of CMB session activities, timing, and CMT activities between sessions. The activities we conducted within sessions were consistent across groups, although we made slight adjustments after group 1, as noted in the description of each session below. For all sessions, a member of the facilitation team took notes and photos to capture all session outputs. Sample agendas with the timing of activities for each session are available in Multimedia Appendix 1.

We did not require collaborators to attend every session, as we anticipated that their busy schedules would not allow this. Collaborators also received a handout with resources for substance use, mental health, and intimate partner violence during the first session they attended. After session 4, we sent out an anonymous feedback survey asking collaborators to rate their level of agreement with statements about the acceptability, feasibility, and appropriateness of session activities (eg, “I liked the activities in each session for this project”). The survey also included open-ended questions (eg, “What did you like the most about the sessions for this project?”). In between groups, the CMT reviewed this feedback to determine if there were ways to improve our approach for future groups. For example, group 2 collaborators said that some of the coloring of session handouts made them hard to read, which we attempted to improve for group 3.

#### CMB Session 1: Variable Elicitation and Ranking

This session began with an overview of the larger CAMPUS project and the overarching goal of the 4 CMB sessions. We provided a definition of sexual violence to ensure a common understanding of our outcome of interest: sexual coercion, nonconsensual sexual contact, and rape [17].

For our first activity, “idea elicitation” (sometimes also referred to as “variable elicitation”), we asked the collaborators to brainstorm the causes and effects of AISV on college campuses, prompting them to think about factors that spanned different social-ecological levels: individual, interpersonal, campus, and societal. We clarified that we were not asking for personal experiences but rather about which factors they thought were most important. The collaborators brainstormed independently before discussing in small groups (maximum 3‐4 people per group). Once they were in small groups, we asked the collaborators to share what they brainstormed, one at a time; put their ideas on sticky notes; and place these sticky notes on a wall. We encouraged the collaborators to combine similar ideas together, to ensure that there was just 1 idea per sticky note and to communicate their idea succinctly. The colors of the sticky notes we provided corresponded to the different social-ecological levels, and we asked the collaborators to match their ideas to these levels using the different colored sticky notes to help ensure that they generated ideas across all 4 levels.

**Figure 2.**
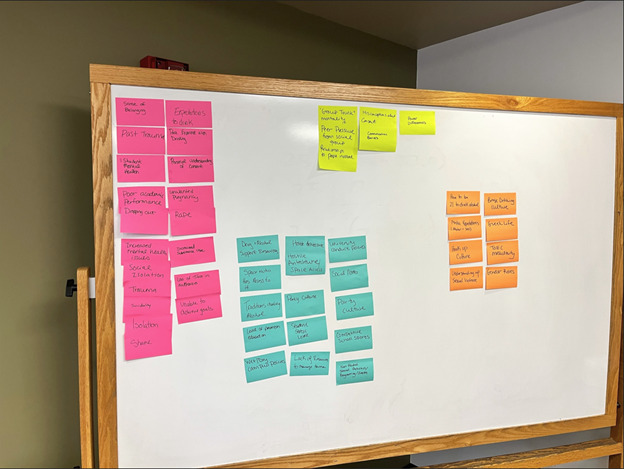
Example of idea elicitation from group 1, session 1.

We designed our second activity, “visualize the vote,” based on human-centered design principles from the Luma Institute [18]. For this activity, we asked the collaborators to walk around the room and review all the ideas posted on the wall by each small group. We gave the collaborators 5 stickers each and asked them to place these stickers on the sticky notes with the 5 most important factors related to AISV on college campuses (Figures2  3). The facilitation team then led a discussion focused on the ideas with the most votes (ie, stickers). For group 1, we asked the collaborators to reflect on which ideas were most important and why. After this session, we found that it would have been more beneficial to spend more time clarifying collaborators’ ideas. Therefore, for groups 2 and 3, we explored whether top-rated ideas represented more than 1 concept (ie, whether they needed to be broken down) or whether there was overlap among ideas (ie, whether any could be combined). In reviewing each top-rated idea with the group, we also asked, “How can we define or unpack this idea?” “Is this idea something that could be modified?” and “Is this something that could increase or decrease?” These last 2 questions were important for transforming these ideas into variables that could be causally linked in subsequent sessions.

**Figure 3.**
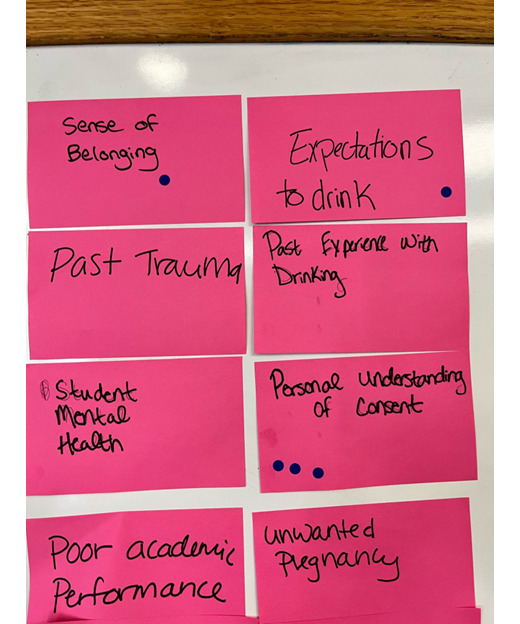
Example of ideas with votes from group 1, session 1.

#### CMT Activities Between Sessions 1 and 2

Using the photos of sticky notes taken during session 1, a member of the CMT transferred all ideas into a Microsoft Excel spreadsheet along with the number of votes for each idea. We developed a curated “short list” of 15 to 25 variables from this list of ideas that could be used for session 2, when collaborators would begin drawing causal connections between these key variables. To create this list, we first grouped the ideas generated by the collaborators into themes and then, within these themes, determined if some ideas needed to be combined (eg, for group 2, we combined “poor mental health resources” and “lack of accessible rehab” into “availability of mental health resources”) and others broken apart (ie, for group 3, we separated “normalization of sex without understanding consent” into “normalization of sex during college” and “understanding of consent and sexual violence”). We then identified the top-rated ideas or variables, taking into account both votes and ideas that were written down more than once. For these top-rated ideas or variables, we refined the phrasing to ensure that each was a modifiable noun that could increase or decrease (eg, we changed “Greek Life” into “percentage of students involved in Greek Life”). The modifiability component was important, as we wanted our final CLD to include leverage points that could be influenced by interventions within the purview of public health and college practitioners. For example, we excluded ideas related to TV or movies and social media that would be difficult to intervene upon for university collaborators. In some cases, we also rephrased ideas such that they were more closely aligned with the research literature; for example, for group 2 we rephrased “hearing other’s good experiences with alcohol/sex” as “expectations that drinking will lead to positive outcomes” and “expectations that sexual experience(s) will lead to positive outcomes,” drawing on our team’s knowledge of alcohol expectancies in the research literature. Aligning collaborators’ ideas with the research literature was important, given our eventual goal of quantifying the CLD into an ABM. However, we acknowledge that modifying the collaborators’ ideas to align with the research literature may have shifted the emphasis away from the collaborator’s perspectives; we attempted to mitigate this by frequently referring back to session notes and following up with participants to ensure that we had a clear understanding of their recommendations. In deciding which variables to include on the curated short list, we first prioritized those that were rated most highly by the collaborators; however, in a small number of cases, we included variables brainstormed by collaborators that received no votes because we thought that they were important based on the literature. The aforementioned example, “expectations that drinking will lead to positive outcomes,” was one such variable.

#### Session 2: Generating Connection Circles

To begin the session, we provided the collaborators with the curated short list of variables on a handout, leaving time to discuss whether any of these variables were unclear to them. We then demonstrated the process of building a connection circle, which are tools for systematically identifying and visualizing the connections between ideas [19]. Beginning with an empty circle drawn on a whiteboard or large piece of paper with our outcome of interest (number of cases of sexual violence) on a sticky note on one part of this circle, we added an idea from the list of variables on another sticky note on the circle. We then asked collaborators to assess whether (1) this newly added variable was a cause or effect of the number of cases of sexual violence and (2) if it increased or decreased (for causes) or was increased or decreased by (for effects) the number of cases of sexual violence. Using markers, we drew an arrow between the 2 variables representing the agreed upon directionality and a plus or minus sign to indicate the type of relationship: a plus sign to indicate if an increase in one variable caused an increase in the other and a minus sign to indicate if an increase in one caused a decrease in the other. We proceeded with adding a second idea and assessing the relationship between this idea, the outcome variable, and the first idea already on the circle.

**Figure 4.**
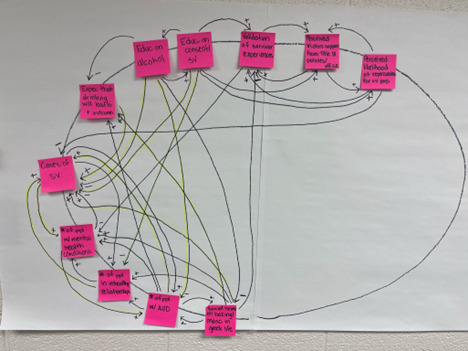
Example connection circle from group 2, session 2.

We then asked the collaborators to break into small groups to complete their own connection circles using the ideas from the short list (Figure 4). We allowed them to add additional ideas not on the list if they wished, reminding them that they should be singular, modifiable nouns that could increase or decrease. We asked them to identify an example of a closed loop with 3 or more ideas, including the number of cases of sexual violence (ie, a loop that started and ended with this variable). Finally, we asked each small group to present an example loop and explain how each of the factors led to an increase or decrease in the other factors.

#### CMT Activities Between Sessions 2 and 3

**Figure 5.**
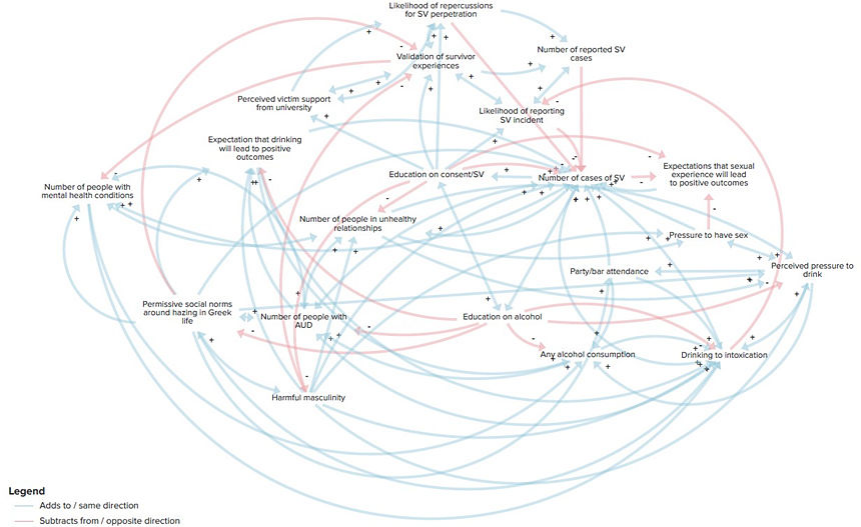
Preliminary causal loop diagram from group 2. AUD: alcohol use disorder; SV: sexual violence.

Using the photos of the connection circles from session 2, a member of the CMT transferred all variables and arrows drawn by the collaborators into Kumu, a freely available online systems mapping platform that enables users to create digital CLDs [20]. To transfer these elements into Kumu, the team member entered the variables from all connection circles into a blank CLD map along with the arrows drawn by the collaborators, creating a preliminary CLD (Figure 5 shows an example of this diagram after session 3 refinements). In a few cases, there were conflicting arrows (ie, one group drew an arrow with a positive sign and another with a negative sign). The CMT identified these arrows, along with other arrows about which they were unsure, for discussion with the collaborators in session 3. The CMT drafted initial definitions for each variable, in some cases, drawing from their knowledge of the research literature (eg, for group 3, we defined “Normalization of drinking during college” as “Descriptive norms: the perception that drinking is acceptable/normal on my campus for people my age”). We were uncertain of how to define some variables and flagged these for discussion in session 3.

#### Session 3: Refining Connection Circles and Intervention Brainstorming

The first half of this session focused on discussing the connections and variables in need of clarification that the CMT had flagged prior to the session. For example, for group 2 for “Number of people in unhealthy relationships,” we presented a spectrum of types of unhealthy relationships based on the Love Is Respect website [21]. The collaborators thought that we should use a broad definition of an unhealthy relationship, including both severe types of unhealthy relationships (eg, abusive behavior) and less severe types (eg, dishonesty and poor communication). As another example, for group 3, we asked the collaborators to explain how increased alcohol accessibility could lead to decreased academic performance or attendance to verify whether another variable (eg, alcohol consumption) belonged on the pathway between these variables. To facilitate these conversations, we provided the collaborators with a printout of our preliminary variable definitions and CLD from Kumu.

The second part of session 3 focused on brainstorming intervention ideas. We asked the collaborators, “Based on our discussion of these variables and this causal loop diagram so far, what intervention would you propose to reduce alcohol-involved sexual violence?” “How would that intervention connect to our existing causal loop diagram?” We prompted the collaborators to think about which variables their proposed intervention would increase or decrease as well as which arrows it would disrupt. We also prompted them to think of interventions free of resource constraints and clarified that interventions could include those that either prevented or reduced the impacts of AISV. After brainstorming independently, the collaborators took turns sharing their ideas with the larger group, explaining how they would influence AISV via the connections in the CLD, and identifying what the audience for each of their proposed interventions would be. We asked about intervention ideas in an open-ended way, as our intention was to elicit potentially novel intervention ideas.

#### CMT Activities Between Sessions 3 and 4

In addition to exploring novel intervention ideas, we wanted collaborator feedback on existing interventions. Thus, as part of our preparation for session 4, we conducted an informal review of peer-reviewed and gray literature to identify established interventions designed to reduce AISV. We wanted collaborators to provide input on the breadth of interventions available; thus, rather than homing in on specific examples, we created a list of intervention types (eg, alcohol-related education, bystander intervention education, and sexual violence victimization reduction). For each intervention type, we identified general intervention objectives and typical formats. If any of the interventions generated by collaborators in session 3 were not already on this list, we added them. During this time, the CMT also implemented updates to variable names and connections on the basis of the input from collaborators during session 3.

#### Session 4: Intervention Ranking

We began session 4 by reviewing any updates to the CLD made on the basis of session 3 discussions and presenting the revised preliminary CLD (see Figure 5 for an example from group 2).

**Figure 6.**
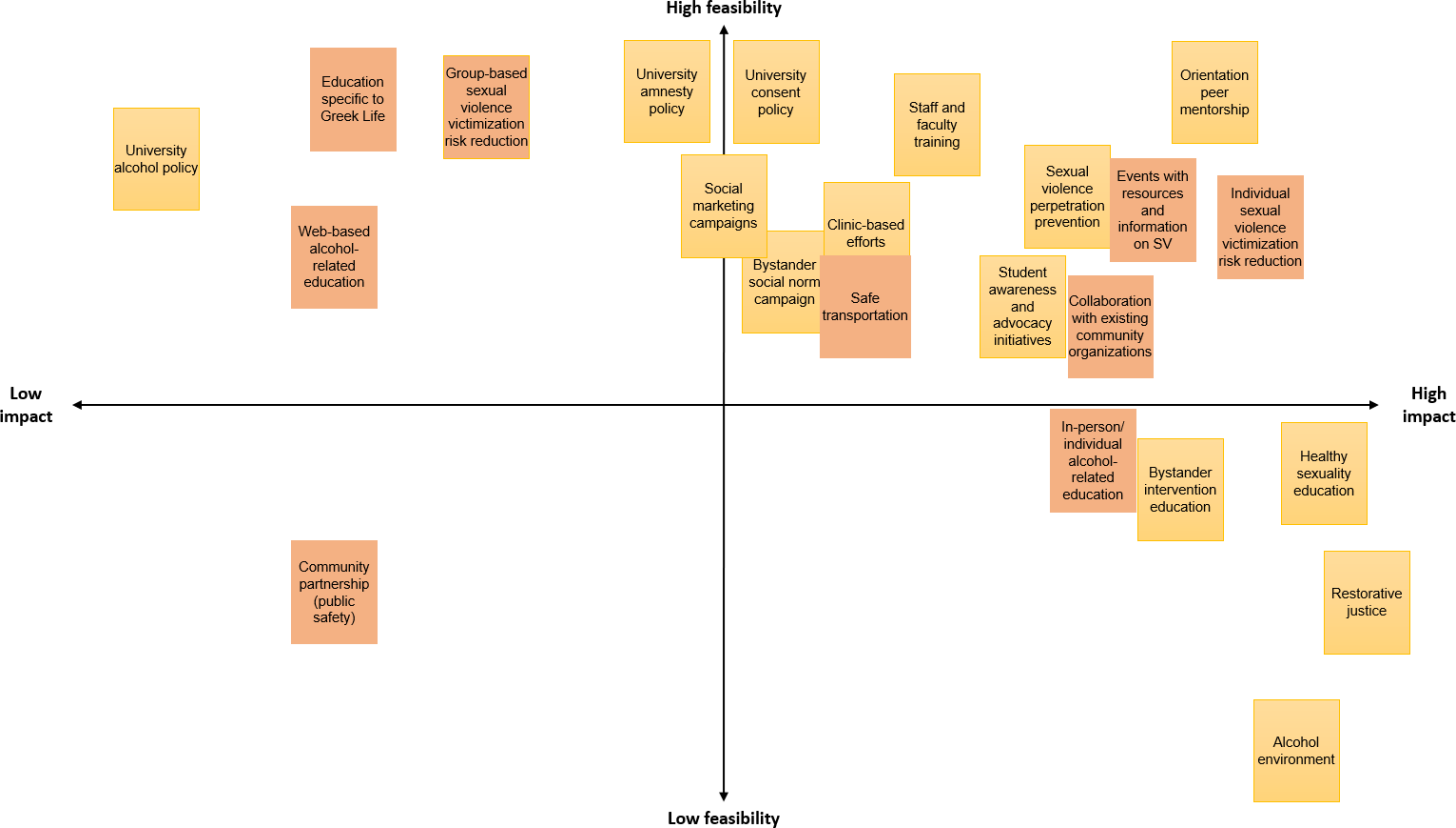
Example of an intervention ranking matrix from group 2. Orange indicates interventions that collaborators added or changed while ranking. SV: sexual violence.

The majority of session 4 was focused on an intervention ranking activity, which we designed on the basis of our experience with human-centered design [22]. The collaborators received a list of intervention types we developed prior to the session (Table S1 in Multimedia Appendix 1). We set up this activity by drawing a matrix on a whiteboard with the y-axis representing high to low levels of feasibility and the x-axis representing high to low levels of impact. We explained our goal to the collaborators: to rank each intervention type on the list provided in terms of how impactful and feasible it might be. Before beginning the activity, we facilitated a brief discussion on what it meant for an intervention to be impactful and feasible to ensure a common understanding of these terms among the collaborators. We then asked the collaborators to take turns picking one intervention type off the list at a time, writing it on a large sticky note, and placing it on the matrix. As more intervention types were selected, collaborators needed to decide the impact and feasibility of each, relative to other intervention types already on the matrix. We encouraged the collaborators to avoid any overlap and prompted the larger group to weigh in on whether they agreed with the placement or ranking. Facilitators guided this conversation to achieve consensus on the ranking of each intervention type. The collaborators continued this process until all intervention types were ranked (Figure 6). We allowed the collaborators to make changes to the intervention types while ranking. For example, group 2 differentiated alcohol-related education into in-person or individual education versus web-based education.

Session 4 ended with a discussion of how the top-ranked interventions could be linked to the CLD. We asked the collaborators, “What loops, connections, or variables might these interventions affect?” “What impact does the intervention have on the number of sexual violence cases and on the overall system?” Our goal with this activity was (1) to understand whether we needed to add any key variables or connections to our CLD and (2) to further understand which mechanisms in the CLD were most important. Ensuring that our final CLD was linked to top-rated interventions was important, given our end goal of translating the CLD into an ABM that could simulate the impacts of various interventions.

### Phase 2: Refinement of Individual CLDs by the CMT

#### Reviewing Variables and Connections

For each CLD, we created an Excel spreadsheet with a row for each arrow in the CLD and columns showing the variable it pointed from, the variable it pointed to, whether the arrow was unidirectional or bidirectional and the sign (+/-) (for the table shell, see Table S2 in Multimedia Appendix 1). We assigned each arrow to 1 of the 3 primary reviewers on the CMT who made initial determinations on the basis of their knowledge of the literature and collaborators’ discussions about if an arrow should be kept, modified, dropped, or investigated further. For the arrows identified as needing further investigation, the primary reviewer identified questions for the larger CMT and next steps for exploring the peer-reviewed literature. We captured the literature review findings in a collaborative Microsoft Word document, discussed questions and findings from literature searches at weekly CMT meetings, and documented final decision points for each arrow in the Excel spreadsheet for each CLD. In general, we sought to ensure that variables were modifiable, potentially measurable, and endogenous to our CLD (ie, modifiable by other variables within the scope of the CLD). We tabled exogenous variables (eg, regulation or oversight of Greek Life from group 3) for considerations as interventions. We kept arrows representing causal relationships that we could substantiate on the basis of empirical evidence, existing theory, or collaborator input. We decided to drop or modify an arrow if the proposed causal connection was too indirect (eg, for group 2, number of people with mental health conditions → individual-level likelihood of drinking to intoxication). In some cases, our review of these arrows led to further refinement of variables and definitions. For example, for group 2, in researching how “pressure to have sex” related to other variables, we found that “hookup culture endorsement” was a commonly used, measurable construct in the research literature that aligned well with the definition discussed with the collaborators during the CMB sessions, and thus we transitioned to using this terminology [23].

Since the CLD for each group came from combining connection circles in which small groups of collaborators had picked different variables from our curated short list (see *Session 2: Generating Connection Circles*), we anticipated that the CLDs may be missing some key connections. Therefore, we also asked the CMT to review the CLD for each group to identify any connections that were missing. In a small number of cases, we added arrows; for example, for group 1, we added an arrow from “number of cases of sexual violence” to “drinking to intoxication.” Some key factors that guided our decision-making throughout this stage included our eventual goal of translating the CLDs into an ABM and whether connections were important from an intervention perspective (ie, discussed as high priority in CMB session 4). Each proposed change to the CLDs was reviewed by at least 2 members of the CMT, including the principal investigator of the study. The output of this stage of this review process was a revised CLD for each group that we then used to identify key loops and narratives, as described below (see Figure S1 in Multimedia Appendix 1).

#### Identifying Loops

The final step in refining the CLD for each group focused on identifying, naming, and describing closed loops (ie, loops that started and ended with the same variable). To do this, each member of the CMT reviewed each CLD and identified 1 to 5 potential closed loops, sometimes proposing the addition of new arrows to make a closed loop. For each proposed loop, we asked the CMT members to provide a potential loop name and narrative (ie, a story describing the dynamics within the loop). During weekly meetings, the entire CMT reviewed each proposed loop. Prior to these conversations, a member of the CMT reviewed all proposed loops, consolidated duplicate suggestions, grouped proposed loops into themes (so that the CMT could discuss similar loops together), and identified and prioritized specific questions for the CMT. Similar to the review of variables and connections described above, we used a collaborative Excel spreadsheet to facilitate this process. For each proposed loop, the spreadsheet included columns capturing the loop name, narrative, connections within each loop, thematic grouping, and key CMT decision points (for the table shell, see Table S3 in Multimedia Appendix 1). If needed, the CMT conducted additional literature searches to justify the addition of connections at this stage.

Kumu enables users to select connections that belong in specific loops, label these loops, and add corresponding narratives. Using this functionality, we transferred all loops identified and adjudicated by the CMT into Kumu. We then deleted variables and connections that were no longer part of any closed loops, archiving them for later review, in case it made sense to reintegrate them in later phases of the project. Finally, we rearranged the CLDs to make these loops more legible and digestible (See Figure S2 in Multimedia Appendix 1). The final output of this phase was a refined CLD with loops labeled and defined for each of the 3 groups.

### Phase 3: CLD Integration

#### Identifying Common Variables Across Groups

To identify the common variables across groups, we first compiled the variables and definitions from all 3 CLDs into a single Excel spreadsheet, noting which CLDs the variable was in (see Table S4 in Multimedia Appendix 1 for the table shell). Next, we grouped the variables into thematic groups to review the variables together and determine if each was conceptually distinct or similar enough to consider combining. For variables identified as being potentially similar enough to combine, we assessed if the changes needed to create a consolidated variable definition would result in substantive changes to the loops in any CLD. For example, we decided to change “alcohol accessibility” from group 3 (“How available is alcohol on/around campus for all students? Number of bars, liquor stores, parties; campus alcohol policies, etc”) to “alcohol availability,” already defined for group 1 as “Is there any alcohol available on a given night/at a given location?” because this change was consistent with the loops already described for group 3. Conversely, we decided to keep “party/bar attendance” from group 2 and “number of parties with alcohol” from group 3 separate, as they represented distinct concepts. To make these changes, a member of the CMT made an initial determination and brought proposed combinations to the larger CMT for discussion and adjudication during weekly meetings. The principal investigator reviewed all determinations prior to implementation.

#### Identifying Common Loops Across Groups

To identify common loops across groups, we created another Excel spreadsheet capturing each loop name, narrative, and the CLD or group from which it came, grouping and sorting the loops thematically (see Table S5 in Multimedia Appendix 1 for the table shell). A member of the CMT reviewed the loops to make an initial determination for each, identifying those that were the exact same (this was the case for only 1 loop), those that were completely distinct, and those that were potentially similar enough to combine. For these loops, we assigned each member of the CMT to 1 set of similar loops and asked them to propose combinations to the larger CMT, which we discussed at weekly meetings. We ultimately decided to combine a small number of loops (4 pairs of loops), which mostly pertained to alcohol availability, norms, and drinking behavior. For newly combined loops, we adjusted the loop narratives to account for changes. We implemented all changes agreed upon by the CMT into a new Kumu project, where we combined the variables, connections, and loops from all 3 CLDs. The output of this phase was a single CLD integrated from each group’s refined CLD (See Figure S3 in Multimedia Appendix 1).

### Phase 4: Finalization of CLD With Collaborator Feedback

#### Conducting Collaborator Feedback Session

In fall 2024, we sent out a web-based Qualtrics survey to the collaborators from all 3 groups via email asking about their interest level in continuing to be involved with the CAMPUS study. Of the 39 collaborators, 20 indicated interest in providing feedback on a refined CLD, 16 indicated interest in working with us to produce dissemination products (eg, one-pagers with study results), and 16 indicated interest in contributing to peer-reviewed manuscripts. Of the 20 who we invited, 12 (7 practitioners and 5 students) attended the feedback session.

The feedback session was 2 hours long, held in an academic conference room and facilitated by the same members of the CMT who facilitated the CMB sessions. We began the session with introductions and a review of the study as well as the activities of the CMT since the end of the CMB sessions (ie, phases 2 and 3 described above). We briefly showed the integrated CLD (Figure S3 in Multimedia Appendix 1) but did not review it in detail, as we reasoned it would be too complex and time-consuming. Instead, we chose to present the specific loops on which we wanted collaborator feedback (11 loops in total, grouped into 5 thematic sections). To do this, we developed a form that presented each of these selected loops, their narratives, and two questions: (1) “What is your overall impression of the loops and narratives above? How accurate or realistic are they?” and (2) “Should anything about the diagram above be changed (ie, should any variables or arrows be removed, added, or modified)?” The form included a refresher on how to read CLDs and space for the collaborators to provide their responses to these questions (Multimedia Appendix 1). We also provided the collaborators with a handout with the variables in the integrated CLD along with their definitions and reminded them to refer to these definitions when reviewing the loops.

For each of the 5 thematic sections, we gave the collaborators time to fill out this form individually, encouraging them to ask their neighbors and the facilitation team clarifying questions about the loops. We then facilitated a group discussion, asking the collaborators to share their feedback on the loops in that section. We chose to break up the discussion like this to facilitate better recall for 2 to 3 loops at a time. We provided hard copies of the feedback form for the collaborators to fill out with pens or pencils but also encouraged them to bring their laptops to the session to fill out a Microsoft Word version of the form, which had been emailed to them ahead of the session. A member of the facilitation team took notes on all discussions. We compensated the collaborators with US $75 each for attending the session, in addition to US $15 to cover parking and transportation costs.

The feedback that we received on one of the loops was inconclusive; thus, in December 2024, we held a one-on-one follow-up conversation over Zoom with 1 collaborator, a practitioner with extensive knowledge and experience in this area. We revised the loop using feedback from this conversation in combination with feedback received from the other collaborators during the feedback session.

#### Integrating Feedback and Quality-Checking the Final CLD

After the session, a member of the CMT compiled all feedback and summarized the key recommendations for each loop. The CMT then reviewed this feedback and decided which changes to make to the CLD to integrate this feedback. For example, based on collaborator feedback, we decided to remove a loop around bystander intervention and campus drinking culture because it showed that alcohol-related problems led to a decrease in positive peer support and connectedness, which many collaborators questioned. Removing this loop meant removing alcohol-related problems entirely from the CLD, which the CMT agreed with since there was substantial overlap between the definition of this variable and that of the outcome (number of cases of sexual violence). In other cases, we made small changes to retained loops and adjusted variable definitions on the basis of collaborator feedback.

**Figure 7.**
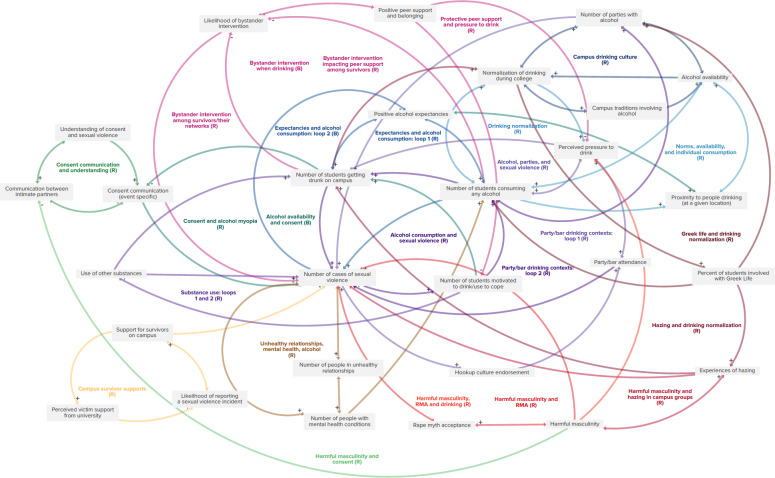
Finalized integrated causal loop diagram. RMA: rape myth acceptance.

Once all changes from the feedback session were implemented by the CMT in January 2025, the CMT conducted final quality checks. To do this, 1 member of the CMT reviewed all loops against prior versions and decision points from all 4 phases and compared the arrows in each loop to the narrative for each loop to make sure they were aligned. This process resulted in a few minor changes to ensure alignment. We also decided to consolidate some drinking variables that existed across different ecological levels (eg, individual-level alcohol consumption vs campus-level alcohol consumption), retaining just the campus-level constructs, as these were more consistent with the narratives within the CLD. The principal investigator reviewed each proposed update, and the larger CMT weighed in on these changes as needed. The output of this step was a finalized version of the integrated CLD (Figure 7).

#### Linking Interventions to Integrated CLD

As a final step, the CMT linked example interventions to the finalized CLD as a proof of concept and to help prioritize which loops to quantify in the next stage of the study. A member of the CMT listed the top 10 intervention types ranked in order of impact from each group during CMB session 4. The CMT grouped these interventions into types based on the thematic category of loops with which they most closely aligned (eg, bystander intervention, alcohol consumption and environment, survivor supports, and harmful masculinity and relationships). For each of these categories, the CMT chose the intervention type ranked most highly in terms of impact (on the basis of the relative positioning of the intervention type on the matrix) across all 3 groups.

For each chosen intervention type, the CMT identified specific intervention examples to more clearly ascertain how intervention end points aligned with variables in the CLD. The CMT turned first to the peer-reviewed literature to identify specific examples, prioritizing those that had been evaluated and had well-described theories of change. In cases where we were not able to identify examples in the literature, we turned instead to feedback from practitioners. For example, because we could not identify an example of Greek Life regulation, we chose to use one of the institutions with Greek Life in our study area as an example. We first conducted a review of the university’s web pages associated with Greek Life. We then held a 1-hour long conversation over Zoom with the Director of Fraternity and Sorority Life at this institution to understand the key elements of how Greek Life was regulated and the potential impacts of these regulations on AISV. Once we had thoroughly described each intervention example, we added it to our CLD by drawing arrows from the intervention to the variables within the CLD based on the intended outcomes of each intervention. If needed, we added interim variables to help illustrate purported causal mechanisms.

## Results

The methods in this paper were implemented, as described, between January 2023 and March 2025. We engaged with 39 campus collaborators across 3 groups to cocreate a CLD with 26 loops containing 28 variables showing key modifiable mechanisms associated with AISV on college campuses. This finalized CLD is the main output of the first aim of the CAMPUS study and will be used to develop an ABM of alcohol-involved sexual violence on college campuses (aim 2) and explore the use of this model as a decision support tool for optimizing interventions to address this issue (aim 3). Analysis of results from the CAMPUS study, funded from September 2022 to June 2027, is ongoing, and results from aim 1 of the study are expected to be published in late 2026.

## Discussion

Alcohol-involved sexual violence is a pressing and persistent problem on college campuses [24,25]. Addressing this issue requires comprehensive, de-siloed approaches that address multiple facets of this issue simultaneously [26-28]. Involving collaborators with practical experience addressing this issue is important for increasing the usefulness of research products [29]. Participatory systems science approaches are well suited for collaboratively addressing complex problems. Although a CLD aims to quantify collaborators’ perspectives on causal mechanisms to a certain extent (eg, describing how key factors can increase or decrease), it is ultimately qualitative in nature. Few studies have sought to translate CLDs into quantified systems dynamics models and ABMs. This study’s goal of developing an ABM based on the CLD produced as part of aim 1 distinguishes the CAMPUS study from other participatory systems science projects. Thus, our contribution to extant participatory systems modeling research is a detailed, transferable post hoc protocol for cocreating a CLD that can be translated into an ABM to support the decisions of college campuses seeking to address AISV among their students.

In this paper, we describe how we integrated input from 3 groups of collaborators with researcher subject matter expertise and findings from peer-reviewed literature to produce a CLD showing causal mechanisms associated with alcohol-involved sexual violence on college campuses and opportunities for modifying these mechanisms. Collaborator brainstorming and ranking of causes and effects of AISV during CMB session 1 provided the initial starting point for our integrated CLD. In subsequent CMB sessions, collaborators identified key causal connections and identified how interventions would modify these connections. The CMT reviewed all variables and connections between and after the CMB sessions in consultation with peer-reviewed literature. After this review and refinement period, collaborators provided additional feedback on causal mechanisms that the CMT could not validate through peer-reviewed literature or through existing theory.

The goal of cocreating a CLD that could be translated into an ABM influenced our decision-making throughout this process and has several implications. It meant that we spent a long time after the CMB sessions (1) refining the CLDs to ensure that the variables and connections in the CLD aligned with peer-reviewed literature as much as possible to facilitate their subsequent quantification and (2) combining CLDs from all 3 groups to create a single CLD that could serve as the basis for ABM development. This refinement (phases 2 and 3) described above was conducted primarily by the CMT and placed more emphasis on researchers’ perspectives as opposed to collaborators’ perspectives (eg, in how variables were defined or adding and dropping certain variables and arrows). We sought to ensure that collaborators’ perspectives were sufficiently reflected in our final CLD by frequently referring to CMB session notes and holding a feedback session (phase 4, described above).

Identifying intervention opportunities is a common component of GMB and other participatory systems science studies [4,14,30,31]. However, the CAMPUS study is unique in its goal of creating a decision support tool for optimizing interventions based on participatory systems science outputs. It is for this reason that we dedicated substantial time during CMB sessions to brainstorm “novel” interventions, sought collaborator input on the relative feasibility and impact of a curated list of interventions that we developed, and concluded our work by linking these interventions to the final CLD. These activities and our incorporation of human-centered design activities differentiate our approach from other participatory model building approaches.

We acknowledge several limitations to our approach. First, as noted above, our approach may have more heavily emphasized researchers’ perspectives over collaborators’ perspectives in order to produce a CLD that could be translated into an ABM. Second, this study was based in Allegheny County, Pennsylvania, and engaged with collaborators who were students and practitioners at institutions of higher education. Researchers engaging with other populations in different contexts to address other complex health problems may need to modify their approach. We also chose to engage 3 separate groups of collaborators. Other projects may seek to engage fewer or more groups and thus need less or more time to integrate CLDs (ie, the integration step is not necessary for studies engaging with just 1 group). Despite these potential differences, this paper serves as a blueprint for other studies seeking to implement CMB and is particularly well suited for studies similarly seeking to translate their CLDs into ABMs or other quantitative systems science models and use these models as decision support tools to inform intervention implementation.

## Supplementary material

10.2196/92071 Multimedia Appendix 1 Supplementary information regarding sample session agendas; a table showing intervention ranking by collaborators; table shells for causal loop diagram variable and connection review, loop identification, variable combination, and loop combination; figures depicting various versions of the causal loop diagram; and the causal loop diagram feedback form.

10.2196/92071 Peer Review Report 1 Peer review report by CIHB - Community Influences on Health Behavior Study Section, Healthcare Delivery and Methodologies Integrated Review Group (National Institutes of Health, USA).

## Abbreviations

ABM: agent-based model
AISV: alcohol-involved sexual violence
CAMPUS: Collaborative Model Building Project to Understand Sexual Violence
CLD: causal loop diagram
CMB: collaborative model building
CMT: core modeling team
GMB: group model building
