# Supplementary material for: Using Collaborative Model Building to Better Understand the Mechanisms of Alcohol-Involved Sexual Violence on College Campuses: Post Hoc Protocol
Source: JMIR Res Protoc. 2026 Jun 8;15:e92071. doi: 10.2196/92071 (PMC13245643; doi:10.2196/92071)
Supplement: Multimedia Appendix 1 [file resprot-v15-e92071-s001.docx]

**Supplement A. Example CMB Session Agendas**

**Session 1**

1. **Introduction [15 min]**
   1. Research team introductions
   2. Expectations/ground rules
   3. Session 1 agenda overview
   4. Icebreaker/introductions
2. **Project Overview/Goals [5 min]**
   1. Define alcohol-involved sexual violence, scope of “problem”.
   2. Introduce social ecological model (“different levels/layers”—individual, interpersonal, etc.).
3. **Idea Elicitation [40 min]**
   1. Individual brainstorming (including across different levels) **[10 min]**
      1. Provide each participant with a stack of stickies notes containing the four different colors and go over the instructions. Let participants know that if they’re not sure which color their idea falls into, that’s okay! They should just make their best guess.
   2. Participants share in small groups **[30 min]**
      1. Ask participant to form groups of 3-4 (If 5 or fewer, just one group)
      2. Participants each take turns sharing what they’ve brainstormed in a round robin fashion.
      3. As they share, encourage participants to ensure there is just one idea per sticky note and that they combine like ideas into a single sticky note.
      4. Facilitators will listen in on group discussions and offer guidance where they can (e.g., helping them identify clusters, social-ecological levels, or condense similar ideas).
      5. As participant are nearly done with their wall, prompt them to discuss in small groups using questions on slide.
4. **Visualizing the Vote [45 min]**
   1. Voting activity **[15 min]**
      1. Participants can vote on any variable across the two groups
      2. Determine how many stickers to give each participant based on how many ideas have been generated (5 per person)
   2. Discuss using questions on slide **[30 min]**
5. **Wrap-up [15 min]**
   1. Briefly explain that next time we will connect the variables together using an activity called “Connection Circles”

**Session 2**

1. **Introduction [15 min]**
   1. Welcome
   2. Session ground rules and expectations
   3. Icebreaker
   4. Overall project aims
2. **Connection Circle Intro [1hr 15 min]**
   1. Intro to Connection Circles
   2. Defining Outcome of Interest
   3. Session 1 Idea Review
   4. Connection Circle Demo.
   5. Walk through instructions
   6. Break up into groups of 3-4 (can be same groups as session 1)
      1. *One group can work from the connection circle we start as a group and the rest can start using other variables. Encourage them to pick the ideas they think are more important for influencing SV cases*
   7. Each group does activity following instructions
      1. As groups finish up, encourage them to identify a closed loop with SV outcome in it and 3+ ideas
3. **Connection Circle Discussion [20 min]**
4. **Wrap-up [10 min]**

**Session 3**

- **Introduction [5 min]**
  - Welcome and consent
  - Overview of session agenda and goals
- **Review of preliminary CLD [1hr 10 min]**
  - High-level overview of CMT activities between sessions 2 and 3
  - Feedback on preliminary CLD; participants will receive handout with the variables and their working definitions + the ones flagged for discussion
- **Action ideas [30 min]**
  - Show full CLD and ask for preliminary feedback on which types of interventions could influence these elements; give participants a few minutes to brainstorm before discussing as a group.
- **Wrap-up [5 min]**

**Session 4**

- **Introduction [5 min]**
  - Welcome and consent
  - Overview of session agenda and goals
- **Review of CLD Refinements [15 min]**
- **Intervention idea ranking [60 min]**
  - Provide introduction to the interventions list, including how we developed it.
  - Introduce ranking activity & discuss meaning of impact and feasibility
  - One at a time, each participant will select an idea and add it to the wall with a sticky note. Other participants will have the opportunity to weigh in on whether they agree. Continue in a round robin fashion (each participant ranking 4-5 ideas each) until all have been ranked. Facilitators will mediate disagreements over ranking and move the process along (e.g., calling on participants to go next).
- **Connecting intervention ideas to the CLD [30 min]**
  - Group discussion: starting with the intervention(s) ranked most **impactful and feasible**, discuss how to connect this intervention to the Causal Loop Diagram
    - What loops, connections, variables might each intervention affect?
    - What impact does this intervention have on:
      - The overall system?
      - Alcohol-involved sexual violence perpetration?
- **Wrap-up [5 min]**

**Supplement B. Supplemental Tables**

**Table S1. Interventions Ranked by Collaborators**

| **Intervention Type** | **Intervention Objectives** | **Typical Formats** | **Changes made by collaborators** |
| --- | --- | --- | --- |
| Sober spaces and events | Alcohol use/intoxication, number of parties, party culture | Residential and social spaces/options designated as alcohol-free | Added by Group 3 and only ranked by this group |
| Alcohol environment | Alcohol use, intoxication, alcohol-related sexual behaviors | Policy | Group 3 thought of this specifically as server-training |
| Third spaces | Social support, likelihood of bystander intervention, drinking expectations, inclusivity | Entertainment venues/places to socialize that are not connected to party scenes | Added by group 1 participants and only ranked by this group |
| Alcohol-related education | Alcohol-related knowledge, attitudes, behaviors | Web-based, motivational interviewing | Group 2 differentiated this into in-person/individual vs. web-based education; Group 3 similarly differentiated this into synchronous vs. asynchronous |
| Education on alcohol and SV for Greek Life | Same as alcohol-related education and SV perpetration prevention/risk reduction, but tailored to Greek life | Interactive in-person individual and group programs | Generated as an idea during Group 2 ranking |
| Bystander intervention education | Bystander intervention skills, speaking out against social norms that support SV, skills to support survivors, education | In-person bystander intervention skills trainings |  |
| Bystander social norm/marketing campaign | Awareness, likelihood of intervention | Campus-wide mixed-media campaigns |  |
| Orientation peer mentorship | Social support, likelihood of bystander intervention, drinking expectations, resource access, incident disclosure | Peer-led, group check-ins and activities post-orientation | Added by Group 1 participants; ranked by subsequent groups |
| Social marketing campaigns | Consent | Designing and disseminating messaging |  |
| Sexual violence perpetration prevention | Consent knowledge, bystander intervention, normative perceptions, sexual violence perpetration | Interactive web-based and in-person programs |  |
| Healthy sexuality education | Sexual risk behaviors, communication and relationship skills, consent knowledge and skills | Classrooms and workshops for high school and college students; peer education programs; presentations via film and theatre; sexual health liaisons for student organizations |  |
| Sexual violence victimization risk reduction | Sexual victimization, dating behaviors, sexual communication, communication with parents, use of self defense | Interactive in-person individual and group programs | Group 2 differentiated this into individual vs. group education |
| Student awareness and advocacy initiatives | Awareness, likelihood of intervention, SV perpetration, responding compassionately, connecting survivors to resources on campus and in the community | Student organizing and campaigns | Group 3 thought of this intervention in-person |
| University alcohol policy | Campus alcohol use, underage drinking | Policy |  |
| Staff and faculty training | Conversational skills, awareness of resources, how/when to report | In-person group training |  |
| Restorative justice | Accountability, risk of reoffending, safety, healing | Collaborative campus-wide processes that include policy development; circle practices to provide meaningful prevention education; restorative conferences that respond to specific incidents of misconduct; and providing support and accountability for offenders who are returning to campus |  |
| Community partnership | Campus safety, tertiary prevention, holding perpetrators "responsible" | Sexual assault response teams, partnerships across staff, law enforcement, community members | Group 2 differentiated this into community partnerships with public safety vs. collaboration with existing community-based organizations |
| University amnesty policy | Reporting medical emergencies around alcohol, sexual violence | Policy |  |
| Clinic-based efforts to prevent and address sexual violence** | Education, behaviors to reduce risk of alcohol-related sexual violence (including bystander intervention), referral to and use of services for survivors | Clinic-based |  |
| Safe late-night transportation | Safe rides between campuses and off-campus destinations | Resources | Added by Group 2 participants and only ranked by this group |
| Events with Resources/Information on SV | Similar to student awareness/advocacy + social marketing campaigns, but could also include objectives similar to bystander norms, and SV risk reduction | In-person group-based programs | Generated as an idea during Group 2 ranking; not on list going into S4 |
| University consent policy | Active/affirmative consent | Policy |  |
| Institutional accountability process navigation | Accountability, peer support, victim blaming, understanding of consent/sexual violence | App-based or in-person supports to increase accessibility and transparency of processes for holding perpetrators accountable | Proposed by Group 3 and only ranked by this group |
| State sexual assault prevention policy | Standardized requirements for affirmative consent, incident disclosure, incident response, and amnesty policies across college campuses; expanded access to law enforcement | Policy | Only ranked in Group 1* |
| Federal sexual assault prevention policy | Protection of survivors' rights; delivery of sexual assault prevention and education programs across college campuses; standardized processes for incident response | Policy | Only ranked in Group 1* |

*We did not include this as an intervention option for Groups 2 and 3 because they are not modifiable by college campus interventionist and are already in place

**Table S2. Causal Loop Diagram Variable and Connection Review Table Shell**

| **Primary reviewer** | **From**  **(variable)** | **To (variable)** | **Direction** | **Type (+/-)** | **Modify this connection? (Keep as is, Modify, Drop, Not sure)** | **Notes/rationale for keeping dropping OR description of proposed change** | **Status/next steps** | **Has this change been implemented in revised CLD?** (Yes/No) | **Additional Notes from CMT Review** |
| --- | --- | --- | --- | --- | --- | --- | --- | --- | --- |
|  |  |  |  |  |  |  |  |  |  |

**Table S3. Loop Identification Table Shell**

| **Primary**  **reviewer** | **CLD/**  **Group #** | **What variables are in the loop?  Include arrows (-->), if possible.** | **Example loop narrative** | **Are there any changes that would need to be made to CLD to make this loop?** | **Potential loop name** | **Theme** | **Other notes** |
| --- | --- | --- | --- | --- | --- | --- | --- |
|  |  |  |  |  |  |  |  |

**Table S4. Variable Combination Table Shell**

| **Variable**  **Theme** | **Variable Name** | **Variable Definition** | **CLD 1** | **CLD 2** | **CLD 3** | **Should this variable be combined with another?** | **If Yes, which variable?** | **Did we decide to keep these separate or combine?** | **If combine, what is the combined variable name?** | **Notes** |
| --- | --- | --- | --- | --- | --- | --- | --- | --- | --- | --- |
|  |  |  |  |  |  |  |  |  |  |  |

**Table S5. Loop Combination Table Shell**

| **Loop theme** | **Loop name** | **CLD** | | | **Should this loop be combined with another?** | **If Yes, which loop (CLD)?** | **Description of combined loop (note any differences that would need to be made to combine)** | **Final decision: Combine (Y/N?** | **Added to combined CLD?** | **Notes** |
| --- | --- | --- | --- | --- | --- | --- | --- | --- | --- | --- |
|  |  | **1** | **2** | **3** |  |  |  |  |  |  |
|  |  |  |  |  |  |  |  |  |  |  |

**Supplement C. Supplemental Figures**


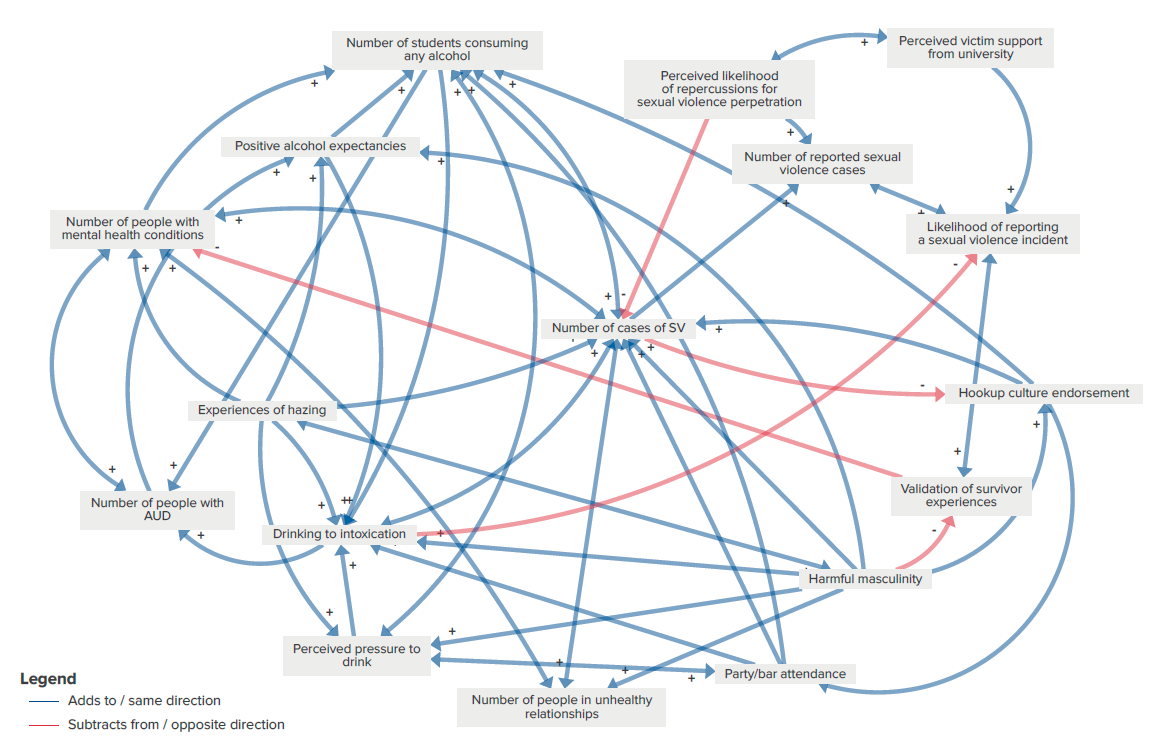


**Figure S1. CLD from Group 2 Revised Following CMT Review**


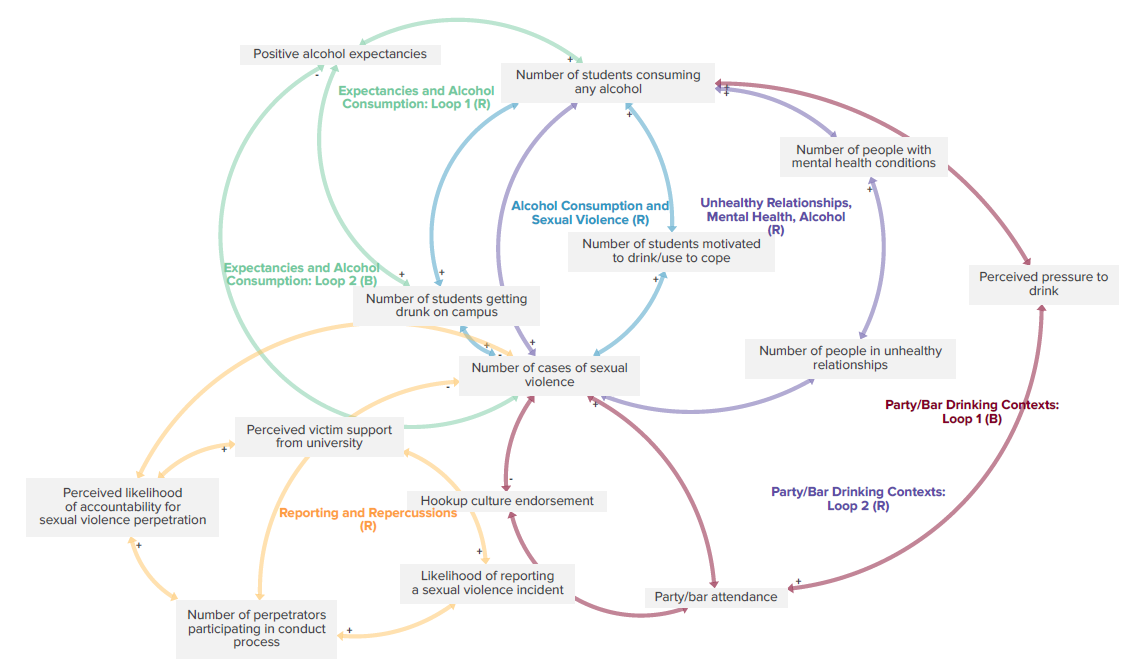


**Figure S2. Final Causal Loop Diagram for Group 2 Following CMT Refinement**


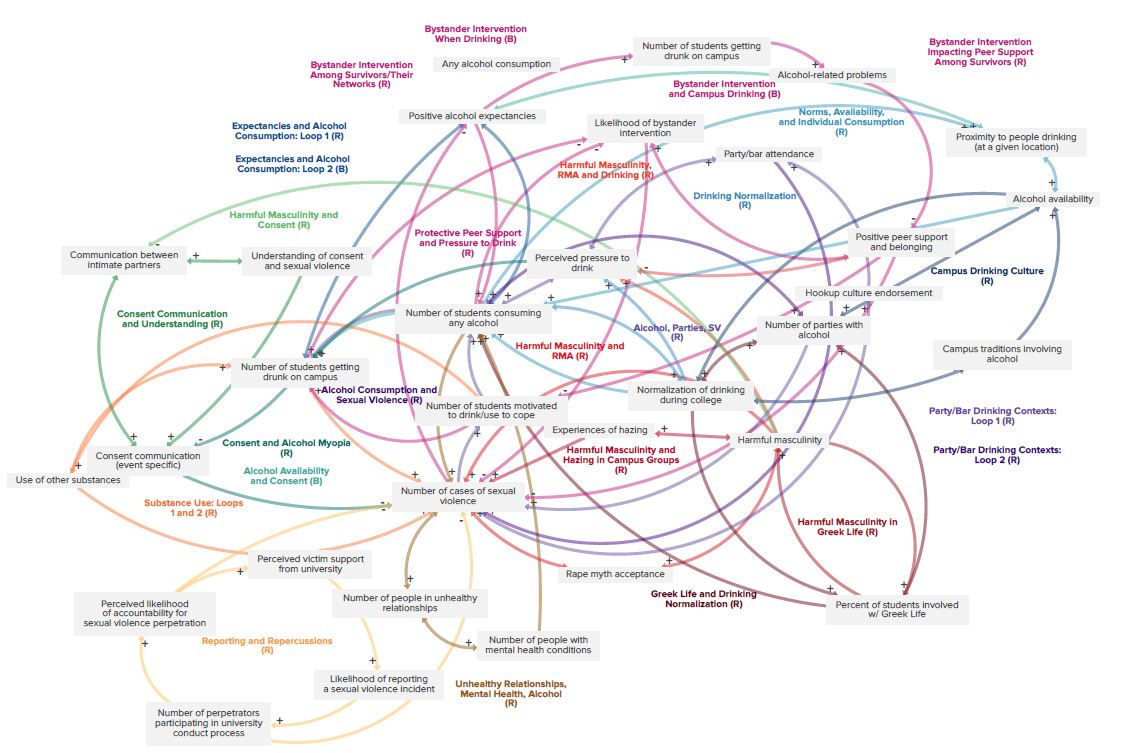


**Figure S3. Initial Integrated CLD**

**Supplement D. Causal Loop Diagram Feedback Form**

**Background:** From April 2023-Feburary 2024, the CAMPUS study met with three groups of collaborators, each of which developed a causal loop diagram illustrating the causes and effects of alcohol-involved sexual violence on college campuses. From February 2024-November 2024 the CAMPUS research team refined and combined these together into one causal loop diagram. During this process, we identified loops about which we were uncertain. The purpose of this form is to seek your input on these loops.

**What are causal loop diagrams again and how do I read them?** Causal loop diagrams illustrate our understanding of how different factors (i.e., variables) cause one another. Arrows with plus signs (+) indicate same direction; an increase in one factor causes an increase in another. Arrows with a negative sign (-) indicate opposite direction; an increase in one factor causes a decrease in another. These loops show how factors can overtime reinforce one another in the same direction (loops labeled with an R are reinforcing loops) or balance each other and maintain stability (loops labeled with a B are balancing loops). We will review examples during the session and answer any questions you may have.

**Instructions for filling out this form:**

- In each of the following sections, review the loops along with their narratives. Refer to the variable definitions sheet as needed.
- Type your answers to the questions below each set of loops
- Work with a neighbor during the session as needed to understand the loops
- Save and email your form with your answers to [redacted]

During the in-person session, we will give you time to fill out this form and will take breaks in between each section to discuss your answers. We may not have time to get to all of the sections during the session, but we still welcome your feedback on all of them via this form.

**Section 1: Harmful Masculinity**

| **Loop Narrative** | **Loop Illustration** |
| --- | --- |
| **Harmful Masculinity and Hazing in Campus Groups:** As the percent of students involved in Greek Life increases, so to do the number of frats and sororities that perpetuate harmful masculine norms. The perpetuation of harmful masculine norms can increase the number of students who might be attracted to such groups, further increasing the number of students involved in Greek Life. In some cases, masculine norms might allow for Greek life organizations to continue or grow (e.g., by allowing for certain power structures to stay in place). Note: harmful masculinity may only increase the % of students in involved in Greek life in some cases, but not others. | 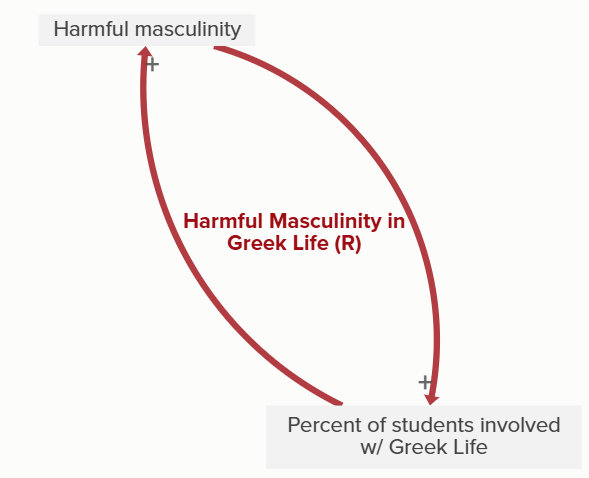 |
| **Harmful Masculinity in Greek Life:** As rape myth acceptance increases, harmful masculinity increases, which causes experiences of hazing to increase, which then increases cases of SV. As cases increase, rape myth acceptance increases, creating a reinforcing loop. | 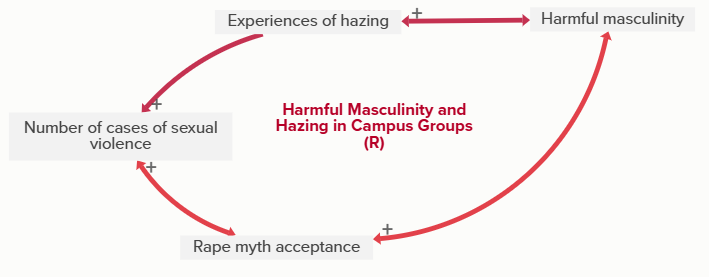 |

**What is your overall impression of the loops and narratives above? How accurate or realistic are they?**

**Should anything about the diagram above be changed (i.e., should any variables or arrows be removed, added, or modified)? Please describe below.**

**During the session we will discuss these loops before moving on to the next ones**

**Section 2: Alcohol Consumption and Drinking Contexts**

| **Loop Narrative** | **Loop Illustration** |
| --- | --- |
| **Party/Bar Drinking Contexts Loop 1:** As more students attend parties/bars, more students feel pressure to drink, increasing the number who drink, and the likelihood of drinking to intoxication. This leads to more cases of alcohol-involved sexual violence, which decreases students' endorsement of hookup culture. This then lowers the number of students attending parties/bars to participate in hookup culture. | 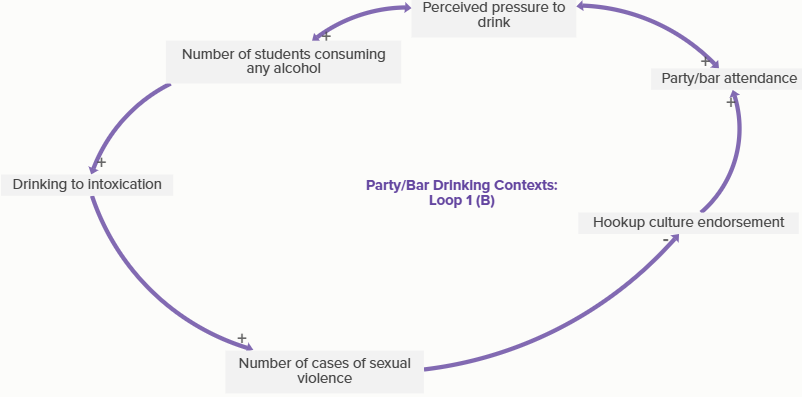 |
| **Party/Bar Drinking Contexts Loop 2:** As cases of SV go up, there's more drinking to cope, which increases the number of students consuming alcohol, increases overall pressure to drink, and increases party/bar attendance. Attending parties/bars in turn increases the number of cases of sexual violence. | 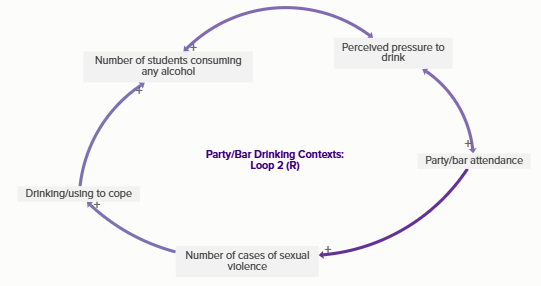 |
| **Alcohol, Parties, SV:** As more students on a campus drink, more parties have alcohol to meet demand. This leads to more cases of SV at/involving parties, which causes more students to drink as a coping mechanism and the total number of students consuming alcohol to increase. | 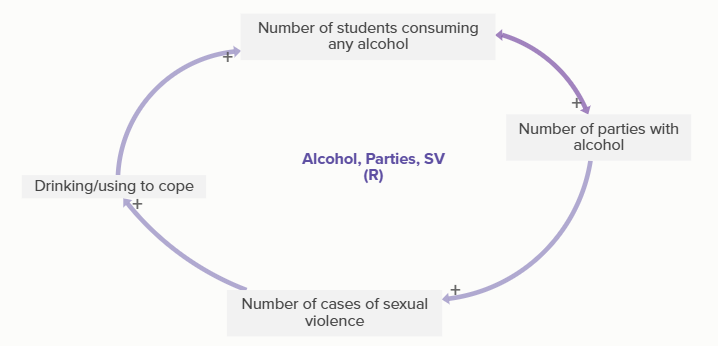 |

**What is your overall impression of the loops and narratives above? How accurate or realistic are they?**

**Should anything about the diagram above be changed (i.e., should any variables or arrows be removed, added, or modified)? Please describe below.**

**During the session we will discuss these loops before moving on to the next ones**

**Section 3: Drinking Culture and Normalization of Drinking**

| **Loop Narrative** | **Loop Illustration** |
| --- | --- |
| **Campus Drinking Culture:** Increased alcohol availability on a campus leads to both increased normalization of drinking during college and more parties with alcohol, which in turn also increases the normalization of drinking during college. The perception that drinking is normal during college further increases campus traditions involving alcohol, which in turn increases alcohol availability. | 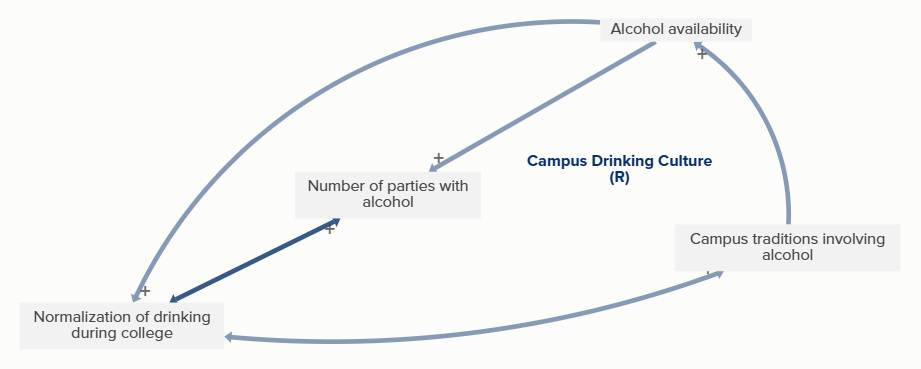 |
| **Norms, Availability, and Individual Consumption:** As the normalization of drinking during college increases, one is more likely to consume alcohol, and therefore be in situations where other people are drinking, increasing proximity to other people drinking, thereby increasing availability of alcohol. This increases the likelihood that one drinks both directly and by further reinforcing the perception that drinking is normal during college. | 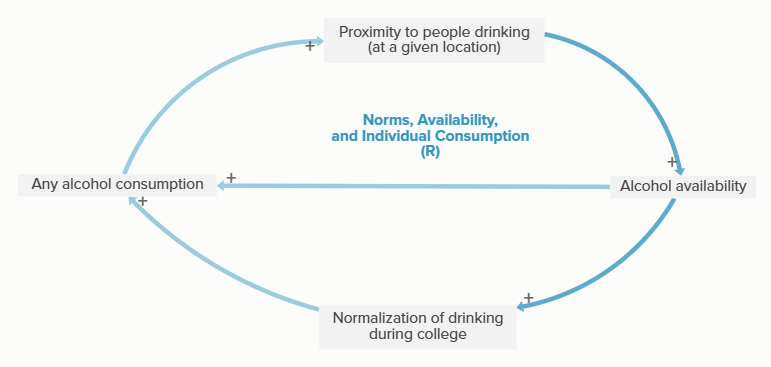 |

**What is your overall impression of the loops and narratives above? How accurate or realistic are they?**

**Should anything about the diagram above be changed (i.e., should any variables or arrows be removed, added, or modified)? Please describe below.**

**During the session we will discuss these loops before moving on to the next ones**

**Section 4: Bystander Intervention and Protective Peer Support**

| **Loop Narrative** | **Loop Illustration** |
| --- | --- |
| **Bystander Intervention Impacting Peer Support:** As positive peer support and connectedness increases, this may decrease the likelihood of cases of SV (e.g., by decreasing likelihood of perpetration), decreasing drinking to cope, which decreases drinking to intoxication. This increases the likelihood of bystander intervention, further strengthening overall positive peer support and connectedness.  A second loop, specific to survivors, is also included here. Increased positive peer support and connectedness can buffer against drinking to cope, decreasing drinking to intoxication, which can increase likelihood of bystander intervention, further strengthening overall positive peer support among survivors. | **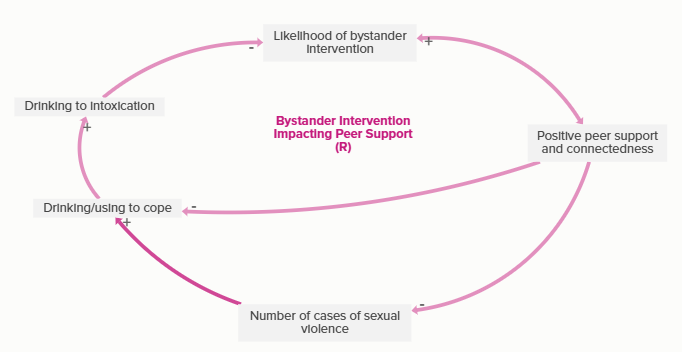** |
| **Protective Peer Support and Pressure to Drink:** As positive peer support and connectedness increase, the pressure to drink decreases, which decreases the extent to which one might drink to intoxication. This increases the likelihood of bystander intervention, further increasing positive peer support and connectedness. | **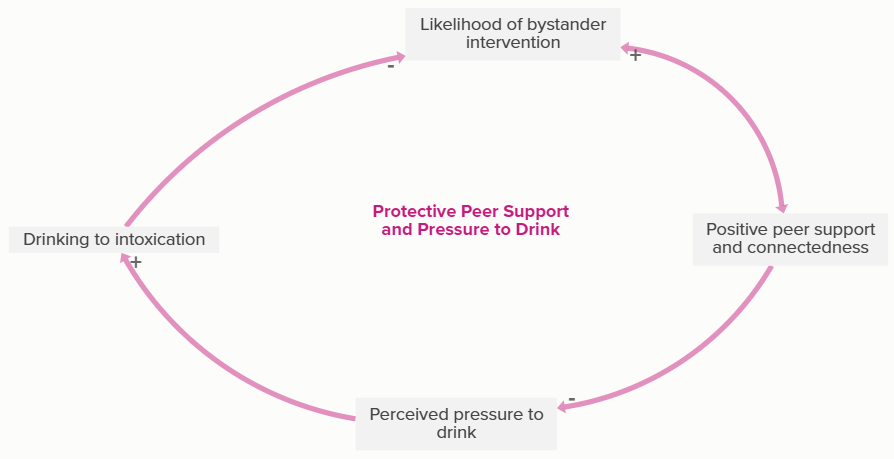** |
| **Bystander Intervention and Campus Drinking:** As the number of students getting drunk on campus increases, the number of problems associated with alcohol use increases, which in turn decreases positive peer support and connectedness. With less positive peer support and connectedness, there is a lower likelihood of bystander intervention, increasing SV cases. As SV cases increase, positive alcohol expectancies go down, and so do the number of students getting drunk. | **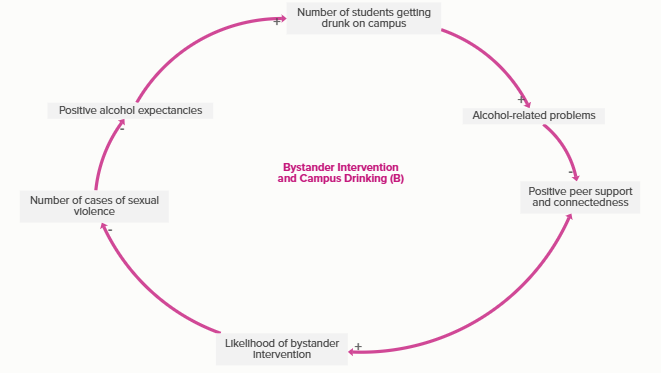** |

**What is your overall impression of the loops and narratives above? How accurate or realistic are they?**

**Should anything about the diagram above be changed (i.e., should any variables or arrows be removed, added, or modified)? Please describe below.**

**During the session we will discuss these loops before moving on to the next ones**

**Section 5: Reporting and Repercussions**

| **Loop Narrative** | **Loop Illustration** |
| --- | --- |
| **Reporting and Repercussions:** As perceived victim support from the university increases, this increases the likelihood of reporting an incident. As more incidents are reported, a higher number of perpetrators are identified and then required to participate in conduct processes, which increases the perceived likelihood of accountability for perpetrators, which in turn increases the perception of victims that the university supports them. As both the perception that perpetrators will be held accountable and perpetrator participation in conduct processes increase, the number of cases of SV will decrease (these last relationships aren’t part of a closed loop). | 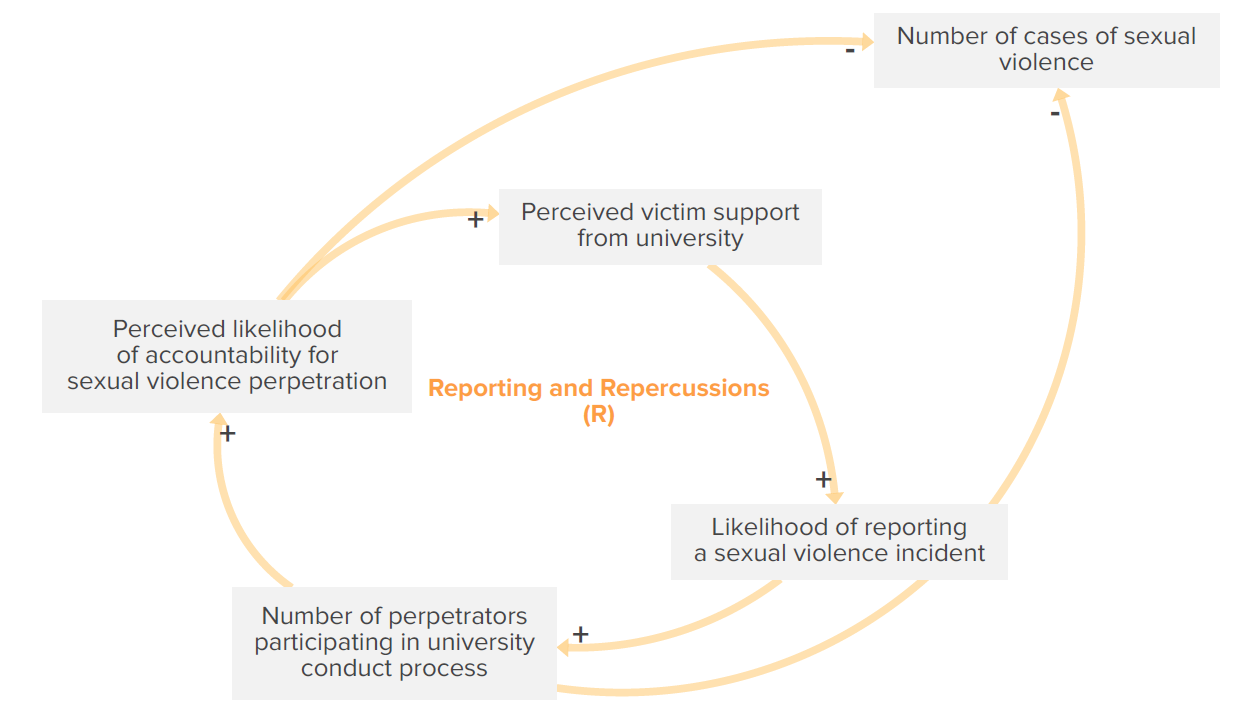 |

**What is your overall impression of the loops and narratives above? How accurate or realistic are they?**

**Should anything about the diagram above be changed (i.e., should any variables or arrows be removed, added, or modified)? Please describe below.**

**Reminder: Please email your completed form to [redacted]!**
