# Supplementary material for: Using Collaborative Model Building to Better Understand the Mechanisms of Alcohol-Involved Sexual Violence on College Campuses: Post Hoc Protocol
Source: JMIR Res Protoc. 2026 Jun 8;15:e92071. doi: 10.2196/92071 (PMC13245643; doi:10.2196/92071)
Supplement: Peer Review Report 1 [file resprot-v15-e92071-s002.pdf]

1R01AA029379-01A1 Mair, Christina

RESUME AND SUMMARY OF DISCUSSION: The goal of this study is to create, validate, and test a stakeholder-engaged agent-based model (ABM) to help college campuses reduce alcohol-related sexual violence. The panel considered the study remarkable and highly innovative. Significance of the study lies in its potential to inform a new type of intervention that could reduce alcohol-related sexual violence on college campuses more effectively. The panel identified several strengths of the application during the discussion, including outstanding team and excellent environment and strong premise and rigorous prior research. The use of human-centered design was noted as particularly innovative and promising. The application has been mostly responsive to the previous comments. The weaknesses noted include a lack of clarity around the calibration process and how all the information from the multiple stakeholder groups will be integrated into the modeling; a lack of information about how and in what circumstances ABM has led to policy change; unclear definition of student stakeholders; and a lack of detail about the qualitative analyses and the decision tool. These weaknesses were considered addressable and do not reduce the project's impact. Overall, the panel agreed the proposed project was very important with a high probability of success.

DESCRIPTION (provided by applicant): Despite increased attention over the past decade, alcohol-involved sexual violence continues to be widespread on college campuses. Existing interventions, which include bystander training, education programs, and brief interventions, take an essentially global approach to the prevention of harmful alcohol use and sexual violence, and largely neglect the social and situational contexts in which drinking occurs. A combination of campus policies, behavioral interventions, and community-level interventions are likely needed to address this complex problem. Alcohol-involved sexual violence can be conceptualized as a system with feedback loops between various individual-level behaviors as well as between contexts and behaviors. Agent-based models (ABMs) are a methodological tool that can incorporate dynamics between behavioral, social, and structural environments and their impacts on hazardous alcohol use and sexual violence. They can be used to simulate the implementation of intervention(s) across a wide range of college campuses and populations. The usefulness of a given ABM, however, is limited by its accuracy, focus, and salience to end users. A stakeholder-engaged model-building process greatly enhances the relevance and usefulness of models that explicitly account for dynamic processes. The overall goal of this proposal is to create a stakeholder-designed ABM that can serve as a decision support tool to help campuses reduce alcohol-involved sexual violence among students. To achieve this goal, we will first develop a stakeholder-designed systems model of hazardous drinking, SV, and intervention opportunities on college campuses through a series of stakeholder-engaged model building sessions guided by a Core Modeling Team (Aim 1). We will leverage a learning collaborative of campus stakeholders including students, practitioners from health and counseling centers, Title IX coordinators, residential counselors, and so forth, from an ongoing alcohol-involved SV RCT involving 28 college campuses in Pennsylvania and West Virginia to recruit 60 student and non-student stakeholder experts to 6 stakeholder model building groups. The integrated systems model will be then translated into an ABM of alcohol use and sexual violence using an ABM platform with realistic synthetic populations that provides a population foundation for community-based simulations (The Framework for Reconstructing Epidemiologic Dynamics (FRED)) (Aim 2). We will then develop and assess the preliminary feasibility of stakeholder-informed, ABM- based decision support tools to optimize campus interventions to reduce alcohol-involved sexual violence (Exploratory Aim 3). This innovative, empirically-based approach, using stakeholder-engaged modeling to design an ABM and ABM-based decision support tools, can guide campus-level policy and programmatic changes, leading to improved implementation of effective strategies to address alcohol-involved sexual violence, promote prevention strategies among students, and stimulate campus-level policy changes.

PUBLIC HEALTH RELEVANCE: Alcohol-involved sexual violence on college campuses may best be addressed through a combination of campus policies and programs, behavioral interventions, and community-level interventions. We will use stakeholder-designed models to create tools to optimize hazardous drinking and sexual violence interventions on college campuses. These efforts will improve implementation of effective strategies, promote prevention strategies among students, and stimulate campus-level policy changes.

CRITIQUE 1

Significance: 1
Investigator(s): 1
Innovation: 1
Approach: 3
Environment: 1

Overall Impact: The goal of this study is to create, validate, and test a stakeholder-engaged agent-based model to help college campuses reduce alcohol-related sexual violence (SV). Across three aims, the study team will create an ABM using a robust stakeholder-engaged process, develop the simulations leveraging an existing modeling environment (FRED), and assess the preliminary feasibility of ABM-based decision support tools to optimize intervention deployment on college campuses. This is an outstanding proposal from a highly qualified scientific team. The project will leverage key existing resources and strengths, including the FRED model, data from a recently completed RCT of over 2,200 students recruited across 28 college campuses, and an engaged stakeholder group from these educational settings. The investigators are ideally positioned and well-prepared to take on this ambitious project, and have demonstrated a successful and productive collaboration. The approach is generally robust but is limited by a few weaknesses, including a poorly integrated conceptual model into the stakeholder-engaged model development process, potential challenges associated with synthesizing one model from six diverse stakeholder groups, and a vague calibration and validation process. Overall, this is a tremendously strong application with high innovation and public health significance and just a few weaknesses in the approach.

1. Significance:

Strengths

- Alcohol-related sexual violence (SV) remains a substantial public health problem, particularly on college campuses. There are limited evidence-based interventions to address alcohol-related SV, and those that do exist primarily target individual and behavioral-level risk factors. This study is significant because it could elucidate new social and structural factors—amenable to intervention—that could more effectively reduce the incidence of alcohol-related SV on college campuses.
- The scientific premise of the proposed work is strong, given the investigators’ previous work in this space and their reliance on FRED, an existing ABM that provides a population foundation for simulation development.
- The project will further benefit from an existing alcohol-involved SV prevention RCT, which further strengthens the scientific premise of the work.

- The likelihood of ABM uptake and utility as a policy-relevant tool is increased through the incorporation of end users throughout a stakeholder-engaged model development process. This is a major strength.

Weaknesses

- None noted by reviewer.

2. Investigator(s):

Strengths

- The principal investigator (Mair) has previously engaged in successful collaborations with the team's co-investigators (publishing over 25 publications together), which is a major strength of the application.
- The principal applicant and other co-investigators have prior experience working with the FRED platform (particularly co-investigator Krauland), which is a major strength.
- The co-investigators Coutler and Burke have experience in employing human-centered design approaches to developing alcohol-related interventions, which is a strength.
- The team's expertise is complementary and the roles are well-delineated, which will increase the likelihood of project success.

Weaknesses

- None noted by reviewer.

3. Innovation:

Strengths

- The use of an ABM to target not one but two points for intervention (e.g., hazardous alcohol use and sexual violence directly) is highly innovative, as these outcomes are usually addressed distinctly and disparately in most studies.
- The focus on human-centered design is an innovative aspect of the study and could push the field forward, as many ABM projects result in end-products with sub-par usability and accessibility.

Weaknesses

- None noted by reviewer.

4. Approach:

Strengths

- The core modeling team (CMT) will identify potential interventions to be included in the model from an existing list of evidence-based interventions, which is a strength.
- The recruitment procedures for Aim 1 are well-described and will leverage an existing relationship with 28 college campuses in Pennsylvania and West Virginia.
- Aim 2 of the study will leverage data from over 2,200 students participating in a large, multi-site RCT focused on alcohol-related SV prevention. This is a major strength of the application, given that the data input needs for an ABM of this complexity are significant.

- The development and testing of multiple decision-support tool prototypes is a strength of the application and will help to ensure that the final product is useful and accessible to the end users.

Weaknesses

- How the conceptual model will inform the stakeholder-engaged model building (SMB) process is not well-described in the application. Will the stakeholders be asked to tackle each level of influence in the socio-ecological model? This is a minor weakness given that the domains to be covered in the first session (SMB) session are described.
- The process and criteria through which the CMT will integrate feedback from six separate groups and synthesize this information into a single model is somewhat under-developed.
- The specific health endpoints and parameter sets to which the model will be calibrated and validated against are not described in the application. This is a moderate weakness.
- Specific calibration procedures are not described beyond a general assessment of model fit. This is another moderate weakness.

5. Environment:

Strengths

- The proposed study will benefit from the resources already in place at the Public Health Dynamics Lab, which manages the FRED infrastructure. This is a major strength.
- Resources are in place to run the agent-based modeling simulations, which can be very resource intensive.

Weaknesses

- None noted by reviewer.

Protections for Human Subjects:

Acceptable Risks and/or Adequate Protections

Data and Safety Monitoring Plan (Applicable for Clinical Trials Only):

Not Applicable (No Clinical Trials)

Inclusion Plans:

- Sex/Gender: Distribution justified scientifically
- Race/Ethnicity: Distribution justified scientifically
- Inclusion/Exclusion Based on Age: Distribution justified scientifically

Vertebrate Animals:

Not Applicable (No Vertebrate Animals)

Biohazards:

Not Applicable (No Biohazards)

Resubmission:

- The investigators have been responsive to the prior critiques and the application is improved as a result.

Resource Sharing Plans:

Acceptable

Budget and Period of Support:

Recommend as Requested

CRITIQUE 2

Significance: 3
Investigator(s): 1
Innovation: 3
Approach: 5
Environment: 1

Overall Impact: The goal of this revised application is to create a stakeholder informed, agent based model (ABM) of the relationship between alcohol use and sexual violence on college campuses. The goal of which is to ultimately drive policy and practice decisions about how to prevent sexual violence on college campuses. Investigators point to gaps in rigour of prior research (that most interventions are behavioral and not consistently effective). The ABM will result in a study of the “universe” of factors that influence alcohol use and sexual violence, including behaviors, practices, policies, and spaces. That is a significant strength of the application. An additional important strength is the inclusion of a wide variety of stakeholders in the ABM design process. Investigators are fabulous – strong track records in this area of research, strong methodological expertise for this innovative study design, and environment has all relevant departments, centers and labs for study to be a success. Boosting feasibility is the embeddedness of this project into an existing study of alcohol and sexual violence on 28 campuses. Approach is aligned to aims and gaps identified in prior research. Many aspects of the approach are clear and reproducible, like stakeholder engagement, data sources, and measures. What is less clear is what ABM looks like in practice. Any examples? What are examples of these decision support tools? It was very hard to visualize what this study will actually produce. Another concern is very little detail about how and in what circumstances ABM has led to policy change. There was one line in prelim studies about a vaccine ABM and changing policies. A literature on the utility of ABM in changing practice would have boosted impact. The study has lots of strengths and addresses an important public health problem. By understanding the universe of determinants of alcohol-related sexual violence, any resulting interventions are likely to have a powerful influence on a field where there are lots of problems but not a lot of good solutions. Enthusiasm was somewhat dampened by noted concerns in approach and for evidence of policy implications.

1. Significance:

Strengths

- A strength of the application is that it considers the universe of determinants of alcohol-related sexual violence on college campuses, including individual behavior, policies, community contexts. That the application uses stakeholders to understand this universe and how it operates will increase its impact and real-world utility to combat a pervasive and terrible public health problem of sexual violence.
- Rigour of prior research is described as investigators point out that much of the work has been in behavioral interventions (not addressing the universe described above) and results have been inconclusive. And that good policies have addressed alcohol use on campus, but not the intersection of alcohol and sexual violence.

Weaknesses

- No evidence was included to show that these ABMs result in actual policy change. Are there examples for when ABMs translate to real change in policy, or outcomes for that matter? A note about driving measles vaccine policy (as a result of ABM) is noted in approach.

2. Investigator(s):

Strengths

- Established PI with long track record of research on agent-based modelling, drinking and sex risk among college students, drinking and intimate partner violence. These experiences are reflected in other NIH grants and pubs. PI well-positioned to carry out present study.
- Lots of expertise on the team in violence prevention, sexually health, community engagement, advanced modeling – all relevant to the current proposal. Division of labor among teammates also seems to be well-articulated.

Weaknesses

- None noted by reviewer.

3. Innovation:

Strengths

- Agent-based modeling is an innovative way to study the universe of determinants of alcohol-involved sexual violence (studying players, contexts, physical spaces).
- Existing technologies (FRED) are being used to deploy the agent based modeling, with examples for how they have previously been successful.
- Stakeholder integration into ABM both an innovation and impact strength

Weaknesses

- Investigators say that ABMs have resulted in policy change but don’t describe how or how they have been effective. This seems critical to be able to judge whether this study will result in major research or clinical paradigm shifts. Is there a small body of research about this?

4. Approach:

Strengths

- Overall, approach / aims designed to address noted gaps in literature

- From a feasibility perspective, embedding this study into an ongoing 28-college project is a strength
- Sampling, stakeholder engagement plans, data source descriptions, measures are clear and these methods replicable
- Problems, alternatives, and sex as a biovariable are described.

Weaknesses

- Stating explicitly why this study is needed above and beyond the parent / previous college campus SV and alcohol study would strengthen rationale. Maybe a table of what is known from the old R01/what remains to be learned from the new R01.
- Hard to see how one could replicate (a score driving issue) the ABM process with the detail provided. What do these sims look like? What are the potential interventions? Examples of ABMs from prior research? Example of a decision support tool, so utility can be assessed?

5. Environment:

Strengths

- Strength of this project is its embeddedness within another R01 – so infrastructure/team for research support already exists.
- Several relevant schools departments and centers are available to support this project: School of public health, department of community health, center for community health, public health dynamics lab. All have expertise within to support team.

Weaknesses

- None noted by reviewer.

Protections for Human Subjects:

Acceptable Risks and/or Adequate Protections

Data and Safety Monitoring Plan (Applicable for Clinical Trials Only):

Not Applicable (No Clinical Trials)

Inclusion Plans:

- Sex/Gender: Distribution justified scientifically
- Race/Ethnicity: Distribution justified scientifically
- Inclusion/Exclusion Based on Age: Distribution justified scientifically

Vertebrate Animals:

Not Applicable (No Vertebrate Animals)

Biohazards:

Not Applicable (No Biohazards)

Resubmission:

- Investigators largely responsive to prior critique - have address theoretical models, have clarified how aims relate to each other, and have addressed other minor comments. Maybe not enough detial still about decision support tools. Dissemination seems fine.

Resource Sharing Plans:

Acceptable

Budget and Period of Support:

Recommend as Requested

CRITIQUE 3

Significance: 2
Investigator(s): 1
Innovation: 2
Approach: 4
Environment: 2

Overall Impact: The proposed work focuses on building an agent-based model to identify potential points of intervention on alcohol related sexual victimization on college campuses. The investigators aim to build decision making tools that can be utilized to effectively intervene on the problem. The proposed work will involve stakeholders (both students and non-students) in the building of agent based models (ABM) that account for multiple levels of influence on the problem of alcohol-related sexual violence, which is a strength of the work. The investigators are experienced and well-situated to conduct the proposed work. They will be building off of prior research and also have experience using the FRED platform. While insufficient details on certain elements of the design impede the assessment of rigor to some degree, the proposed work has the potential for impact if the decision making tool is successfully produced based on the ABM.

1. Significance:

Strengths

- The problem of alcohol related sexual victimization remains a persistent problem on college campuses. It would be significant to identify new ways to intervene through decision making tools.
- The investigators consider a multi-level approach to the problem rather than focusing solely on the individual level.

Weaknesses

- Within the context of the proposal, the socio-ecological model is more a loose framework indicating a need to consider multi-level factors than an actual theory that establishes what may be anticipated.

2. Investigator(s):

Strengths

- The PI is experienced in developing this type of work, in particular with the FRED system, and the proposed work building on this prior work.
- The PI has assembled a multi-disciplinary team of investigators to support the research.

Weaknesses

- None noted by reviewer.

3. Innovation:

Strengths

- The use of stakeholder influenced agent based modeling is a strength of the project's innovation.
- The development of a decision making tool could be an impactful innovation.

Weaknesses

- While the decision making tool is potentially innovative, limited details make it unclear about the extent of the innovation

4. Approach:

Strengths

- The involvement of stakeholders (students and non-students) in building the ABM is a strength of the proposed work.
- The use of the FRED system is a clear strength of the proposed work.
- The study leverages an existing study (GIFTSS)

Weaknesses

- Greater specificity in how student stakeholders are defined would more clearly enhance the rigor of the approach. Are there specific types of students who need to be targeted as stakeholders?
- The details on how the stakeholder sessions would transition to the ABM was not fully clear.
- It is unclear within the application whether there are standards in place for a minimum number of simulations necessary to achieve optimal results with these specific types of models.
- It is unclear whether Aim 2 may be fully complete if the ABM does not include every element of the systems model developed in Aim because it is too computationally complex.
- The qualitative analysis described would benefit from greater detail.
- The development of the decision making tool would have benefited from greater detail.

5. Environment:

Strengths

- The institutional environment provides the appropriate resources and support to carry out the proposed work.

Weaknesses

- None noted by reviewer.

Protections for Human Subjects:
Acceptable Risks and/or Adequate Protections

- The balance of risks and benefits is satisfactory.

Data and Safety Monitoring Plan (Applicable for Clinical Trials Only):
Not Applicable (No Clinical Trials)

Inclusion Plans:

- Sex/Gender: Distribution justified scientifically
- Race/Ethnicity: Distribution justified scientifically
- Inclusion/Exclusion Based on Age: Distribution justified scientifically
- The distribution of the study population is justified scientifically

Vertebrate Animals:
Not Applicable (No Vertebrate Animals)

Biohazards:
Not Applicable (No Biohazards)

Resubmission:

- The applicants were moderately responsive to the prior reviews.

Budget and Period of Support:
Recommend as Requested

THE FOLLOWING SECTIONS WERE PREPARED BY THE SCIENTIFIC REVIEW OFFICER TO SUMMARIZE THE OUTCOME OF DISCUSSIONS OF THE REVIEW COMMITTEE, OR REVIEWERS' WRITTEN CRITIQUES, ON THE FOLLOWING ISSUES:

PROTECTION OF HUMAN SUBJECTS: ACCEPTABLE

INCLUSION OF WOMEN PLAN: ACCEPTABLE

INCLUSION OF MINORITIES PLAN: ACCEPTABLE

INCLUSION ACROSS THE LIFESPAN: ACCEPTABLE

COMMITTEE BUDGET RECOMMENDATIONS: The budget was recommended as requested.

Footnotes for 1 R01 AA029379-01A1; PI Name: Mair, Christina Furber

NIH has modified its policy regarding the receipt of resubmissions (amended applications).See Guide Notice NOT-OD-18-197 at https://grants.nih.gov/grants/guide/notice-files/NOT-OD-18-197.html. The impact/priority score is calculated after discussion of an application by averaging the overall scores (1-9) given by all voting reviewers on the committee and multiplying by 10. The criterion scores are submitted prior to the meeting by the individual reviewers assigned to an application, and are not discussed specifically at the review meeting or calculated into the overall impact score. Some applications also receive a percentile ranking. For details on the review process, see http://grants.nih.gov/grants/peer_review_process.htm#scoring.

MEETING ROSTER

Community Influences on Health Behavior Study Section
Healthcare Delivery and Methodologies Integrated Review Group
CENTER FOR SCIENTIFIC REVIEW
CIHB
10/26/2021 - 10/27/2021

Notice of NIH Policy to All Applicants: Meeting rosters are provided for information purposes only. Applicant investigators and institutional officials must not communicate directly with study section members about an application before or after the review. Failure to observe this policy will create a serious breach of integrity in the peer review process, and may lead to actions outlined in NOT-OD-14-073 at https://grants.nih.gov/grants/guide/notice-files/NOT-OD-14-073.html, NOT-OD-15-106 at https://grants.nih.gov/grants/guide/notice-files/NOT-OD-15-106.html, and NOT-OD-18-115 at https://grants.nih.gov/grants/guide/notice-files/NOT-OD-18-115.html, including removal of the application from immediate review.

CHAIRPERSON(S)

RAMASWAMY, MEGHA, MPH, PHD
PROFESSOR
DEPARTMENT OF POPULATION HEALTH
UNIVERSITY OF KANSAS SCHOOL OF MEDICINE
KANSAS CITY, KS 66160

MEMBERS

BEAMER, PALOMA I, PHD
ASSOCIATE PROFESSOR
DEPARTMENT OF COMMUNITY, ENVIRONMENT AND POLICY
MEL AND ENID ZUCKERMAN COLLEGE OF PUBLIC HEALTH
UNIVERSITY OF ARIZONA
TUCSON, AZ 85724

BOCH, SAMANTHA J, PHD *
ASSISTANT PROFESSOR
COLLEGE OF NURSING
UNIVERSITY OF CINCINNATI
CINCINNATI, OH 45221

BONAR, ERIN ELIZABETH, PHD
ASSOCIATE PROFESSOR
DEPARTMENT OF PSYCHIATRY
UNIVERSITY OF MICHIGAN
ANN ARBOR, MI 48109

BORRELL, LUISA N, DDS, PHD *
DISTINGUISHED PROFESSOR
DEPARTMENT OF EPIDEMIOLOGY AND BIOSTATISTICS
GRADUATE SCHOOL OF PUBLIC HEALTH
AND HEALTH POLICY
CITY UNIVERSITY OF NEW YORK
NEW YORK, NY 10027

CLARK, TRENETTE T, PHD *
SANDRA REEVES SPEARS AND JOHN B. TURNER
DISTINGUISHED PROFESSOR
SCHOOL OF SOCIAL WORK
THE UNIVERSITY OF NORTH CAROLINA AT CHAPEL HILL
CHAPEL HILL, NC 27599

CORLISS, HEATHER L, PHD
PROFESSOR
DIVISION OF HEALTH PROMOTION
AND BEHAVIORAL SCIENCE
SCHOOL OF PUBLIC HEALTH
SAN DIEGO STATE UNIVERSITY
SAN DIEGO, CA 92123

CUNRADI, CAROL B, PHD *
SENIOR RESEARCH SCIENTIST
PREVENTION RESEARCH CENTER
PACIFIC INSTITUTE FOR RESEARCH AND EVALUATION
BERKELEY, CA 94704

DUNCAN, GLEN E, PHD
PROFESSOR AND CHAIR
DEPARTMENT OF NUTRITION AND EXERCISE PHYSIOLOGY
ELSON S. FLOYD COLLEGE OF MEDICINE
WASHINGTON STATE UNIVERSITY
SPOKANE, WA 99202

FREISTHLER, BRIDGET J, PHD *
PROFESSOR
COLLEGE OF SOCIAL WORK
THE OHIO STATE UNIVERSITY
COLUMBUS, OH 43210

GOHLKE, JULIA M, PHD
ASSOCIATE PROFESSOR
DEPARTMENT OF POPULATION HEALTH SCIENCES
VIRGINIA POLYTECHNIC INSTITUTE
AND STATE UNIVERSITY
BLACKSBURG, VA 24061

GOODKIND, JESSICA R, PHD
ASSOCIATE PROFESSOR
DEPARTMENT OF SOCIOLOGY
UNIVERSITY OF NEW MEXICO
ALBUQUERQUE, NM 87131

HAHN, ELLEN J, PHD, DNS *
PROFESSOR
COLLEGE OF NURSING
UNIVERSITY OF KENTUCKY
LEXINGTON, KY 40502

HAN, BING, PHD *
SENIOR RESEARCH SCIENTIST
DEPARTMENT OF RESEARCH AND EVALUATION
KAISER PERMANENTE
PASADENA, CA 91101

HIRATSUKA, VANESSA Y, PHD
ASSISTANT PROFESSOR AND CO-DIRECTOR
CENTER FOR HUMAN DEVELOPMENT
UNIVERSITY OF ALASKA ANCHORAGE
ANCHORAGE, AK 99508

KELLY, BRIAN C, PHD *
ASSOCIATE PROFESSOR
DEPARTMENT OF SOCIOLOGY
PURDUE UNIVERSITY
WEST LAFAYETTE, IN 47907

LELUTIU-WEINBERGER, CORINA, PHD *
ASSOCIATE PROFESSOR AND ENDOWED CHAIR OF THE
FRANÇOIS-XAVIER BAGNOUD CENTER
RUTGERS BIOMEDICAL AND HEALTH SCIENCES
SCHOOL OF NURSING
RUTGERS UNIVERSITY
NEWARK, NJ 07101

MARSHALL, BRANDON DAVID LEWIS, PHD
ASSOCIATE PROFESSOR
DEPARTMENT OF EPIDEMIOLOGY
BROWN UNIVERSITY SCHOOL OF PUBLIC HEALTH
PROVIDENCE , RI 02912

MASON, MICHAEL JON, PHD
BETSEY R BUSH ENDOWED PROFESSOR
CENTER FOR BEHAVIORAL HEALTH RESEARCH
COLLEGE OF SOCIAL WORK
UNIVERSITY OF TENNESSEE
KNOXVILLE, TN 37996

MASSEY, PHILIP M, PHD
ASSOCIATE PROFESSOR
DEPARTMENT OF HEALTH, HUMAN PERFORMANCE
AND RECREATION
UNIVERSITY OF ARKANSAS
FAYETTEVILLE, AR 72701

MOJTABAI, RAMIN, PHD, MD
PROFESSOR
DEPARTMENT OF MENTAL HEALTH
BLOOMBERG SCHOOL OF PUBLIC HEALTH AND
DEPARTMENT OF PSYCHIATRY AND BEHAVIORAL
SCIENCES
JOHNS HOPKINS UNIVERSITY
BALTIMORE, MD 21205

PAHL, KERSTIN, PHD
RESEARCH ASSOCIATE PROFESSOR
DEPARTMENT OF PSYCHIATRY
NEW YORK UNIVERSITY SCHOOL OF MEDICINE
NEW YORK, NY 10016

PERKINS, MOLLY M, PHD *
ASSOCIATE PROFESSOR
DIVISION OF GERIATRICS AND GERONTOLOGY
DEPARTMENT OF MEDICINE
EMORY UNIVERSITY SCHOOL OF MEDICINE
ATLANTA, GA 30322

QUINTILIANI, LISA M, PHD
ASSOCIATE PROFESSOR
SCHOOL OF MEDICINE
BOSTON UNIVERSITY
BOSTON, MA 02118

ROY, BRITA, MD *
ASSISTANT PROFESSOR OF MEDICINE
DEPARTMENT OF INTERNAL MEDICINE
DIRECTOR OF POPULATION HEALTH
SCHOOL OF MEDICINE
YALE UNIVERSITY
NEW HAVEN, CT 06510

RUDOLPH, ABBY ELISE, BA, MPH, PHD *
ASSOCIATE PROFESSOR
COLLEGE OF PUBLIC HEALTH
TEMPLE UNIVERSITY
PHILADELPHIA, PA 19122

SCARINCI, ISABEL C, PHD
PROFESSOR
DIVISION OF PREVENTIVE MEDICINE
DEPARTMENT OF MEDICINE
UNIVERSITY OF ALABAMA AT BIRMINGHAM
BIRMINGHAM, AL 35294

SHARMA, SHREELA V, PHD *
PROFESSOR
DIVISION OF EPIDEMIOLOGY
ENVIRONMENTAL HEALTH AND GENETICS
SCHOOL OF PUBLIC HEALTH
UNIVERSITY OF TEXAS
HOUSTON, TX 77030

SMART, MIEKA JASMINE, DRPH *
ASSISTANT PROFESSOR
DEPARTMENT OF EPIDEMIOLOGY AND BIOSTATISTICS
DIVISION OF PUBLIC HEALTH
COLLEGE OF HUMAN MEDICINE
MICHIGAN STATE UNIVERSITY
FLINT, MI 48502

THRASHER, JAMES, PHD
PROFESSOR
DEPARTMENT OF HEALTH PROMOTION,
EDUCATION AND BEHAVIOR
ARNOLD SCHOOL OF PUBLIC HEALTH
UNIVERSITY OF SOUTH CAROLINA
COLUMBIA, SC 29208

WARD, TONY JOHN, PHD
PROFESSOR AND CHAIR
SCHOOL OF PUBLIC
AND COMMUNITY HEALTH SCIENCES
UNIVERSITY OF MONTANA
MISSOULA, MT 59812

WIEHE, SARAH ELIZABETH, MD, MPH
ASSOCIATE PROFESSOR
DEPARTMENT OF PEDIATRICS
CHILDREN'S HEALTH SERVICES RESEARCH
INDIANA UNIVERSITY SCHOOL OF MEDICINE
INDIANAPOLIS, IN 46202

WILLIAMS, EDITH MARIE, PHD
ASSOCIATE PROFESSOR
DIVISION OF RHEUMATOLOGY
DEPARTMENT OF PUBLIC HEALTH SCIENCES
MEDICAL UNIVERSITY OF SOUTH CAROLINA
CHARLESTON, SC 29425

MAIL REVIEWER(S)

TASDIZEN, TOLGA, BS, MS, PHD
ASSOCIATE PROFESSOR
DEPARTMENT OF ELECTRICAL AND
COMPUTER ENGINEERING
UNIVERSITY OF UTAH
SALT LAKE CITY, UT 84112

SCIENTIFIC REVIEW OFFICER

PELTOLA, PIA KRISTIINA, PHD
SCIENTIFIC REVIEW OFFICER
CENTER FOR SCIENTIFIC REVIEW
BETHESDA, MD 20892

EXTRAMURAL SUPPORT ASSISTANT

JONES, BELINDA
EXTRAMURAL SUPPORT ASSISTANT
CENTER FOR SCIENTIFIC REVIEW
NATIONAL INSTITUTES OF HEALTH
BETHESDA, MD 20892

* Temporary Member. For grant applications, temporary members may participate in the entire meeting or may review only selected applications as needed.

Consultants are required to absent themselves from the room during the review of any application if their presence would constitute or appear to constitute a conflict of interest.
